# Verteporfin disrupts multiple steps of autophagy and regulates p53 to sensitize osteosarcoma cells

**DOI:** 10.1186/s12935-020-01720-y

**Published:** 2021-01-14

**Authors:** Heena Saini, Harshita Sharma, Sudeshna Mukherjee, Shibasish Chowdhury, Rajdeep Chowdhury

**Affiliations:** Department of Biological Sciences, BITS Pilani, Pilani Campus, Rajasthan, 333031 India

**Keywords:** Osteosarcoma, Verteporfin, Autophagy, Proteasome inhibitor, P53

## Abstract

**Background:**

Osteosarcoma (OS) is a malignant tumor of the bone mostly observed in children and adolescents. The current treatment approach includes neoadjuvant and adjuvant chemotherapy; however, drug resistance often hinders therapy in OS patients. Also, the post-relapse survival of OS patients is as low as 20%. We therefore planned to understand the molecular cause for its poor prognosis and design an appropriate therapeutic strategy to combat the disease.

**Methods:**

We analyzed OS patient dataset from Gene Expression Omnibus (GEO) and identified the differentially expressed genes and the top deregulated pathways in OS. Subsequently, drugs targeting the major de-regulated pathways were selected and the following assays were conducted- MTT assay to assess cytotoxicity of drugs in OS cells; immunoblotting and immunostaining to analyze key protein expression and localization after drug treatment; LysoTracker staining to monitor lysosomes; Acridine Orange to label acidic vesicles; and DCFDA to measure Reactive Oxygen Species (ROS).

**Results:**

The differential gene expression analysis from OS patient dataset implicated the striking involvement of cellular processes linked to autophagy and protein processing in the development of OS. We therefore selected the FDA approved drugs, chloroquine (CQ) and verteporfin (VP) known for autophagy inhibitory and proteotoxic functions to explore against OS. Importantly, VP, but not CQ, showed an extensive dose-dependent cytotoxicity. It resulted in autophagy disruption at multiple steps extending from perturbation of early autophagic processes, inhibition of autophagic flux to induction of lysosomal instability. Interestingly, VP treated protein lysates showed a ROS-dependent high molecular weight (HMW) band when probed for P62 and P53 protein. Further, VP triggered accumulation of ubiquitinated proteins as well. Since VP had a pronounced disruptive effect on cellular protein homeostasis, we explored the possibility of simultaneous inhibition of the ubiquitin-proteasomal system (UPS) by MG-132 (MG). Addition of a proteasomal inhibitor significantly aggravated VP induced cytotoxicity. MG co-treatment also led to selective targeting of P53 to the lysosomes.

**Conclusion:**

Herein, we propose VP and MG induce regulation of autophagy and protein homeostasis which can be exploited as an effective therapeutic strategy against osteosarcoma.

## Background

Osteosarcoma (OS) is an aggressive malignancy of the bone, which is predominantly observed in children and adolescents [1]. The current treatment approach for OS often includes a multi-modal therapeutic procedure with a pre-operative poly-chemotherapy followed by surgery, and then a post-operative chemotherapy again [2]. However, even with such an aggressive treatment regime, chemotherapy often fails in patients with OS. Also, there is no standard therapeutic regimen for OS patients, with a relapse of disease conditions [3]. This despondency demands further careful analysis of the molecular signature of OS and design of an appropriate therapeutic strategy to tame the disease.

In this context, analysis of the existing literature indicates a plausible role of the cellular homeostatic process- autophagy in OS pathogenesis [4–7]. Autophagy is conventionally known to be involved in recycling of cellular macromolecules and damaged organelles, thus maintaining cellular homeostasis. During this process, the cargoes are typically engulfed in membrane bound vesicles, known as autophagosomes, followed by their fusion with lysosomes for subsequent degradation. However, the role of autophagy in cancer is controversially discussed over literature. It is reported to mediate contrasting cellular effects, like, cell survival or cell death in a context dependent manner. This implicates the need for further exploration of the role of autophagy in cancer [8]. In osteosarcoma cells, for example, an inhibition of autophagy, with the early autophagy inhibitor, 3-methyladenine (3-MA) was found to increase paclitaxel-induced apoptotic cell death in Saos-2 cells [9]. In another study, an HSP90AA1 and HMGB1-mediated autophagy was found to induce drug resistance in OS cells [4, 5]. Importantly, cumulative evidence indicates a probable pro-survival role of autophagy in OS cells. However, further research in this direction is required to enable introduction of a prospective treatment strategy, targeting autophagy, against cancers like OS.

Earlier studies by Guo et al. describes the importance of P53 in the development of OS [10]. P53 protein is typically known for its tumor suppressive role. However, it is mutated in more than half of the cancers. Depending on the locus, a vast majority of these mutations not only can nullify P53 proteins’ tumor-suppressive function but can instead impart a tumor promoting effect. Such mutations leading to acquisition of new properties are often known as gain of function (GOF) mutations [11, 12]. Interestingly, the overall frequency of P53 mutation in people with osteosarcoma is as high as 22% [13, 14]. In OS cells as well, while the wild type-P53 (WT-P53) protein increases chemo-sensitivity [15], contrastingly, the GOF-mutant-P53 mediates chemo-resistance [16]. Therefore, GOF-mutations in P53 imposes an eminent problem in OS sensitization and remains a significant challenge to address. P53 protein is also known to have considerable crosstalk with autophagy and may thus have critical inter-regulatory functions [17, 18]. Hence, we apprehend that molecules that can modulate key cellular processes like autophagy and can simultaneously neutralize GOF-mutant induced effects might be critical to a successful OS therapy.

In this study, we analyzed differential gene expression data from the GEO database. Autophagy associated processes were found to be highly dysregulated in OS. Subsequently, we re-purposed the FDA approved drugs, CQ or VP, to inhibit autophagy and explore their potential in sensitizing OS cells. While CQ did not show a significant cytotoxic effect, VP was predominantly effective in disrupting multiple steps of autophagy and was cytotoxic to the OS cells. An enhanced cytotoxic effect was observed with addition of a proteasomal inhibitor. VP also caused an accumulation of HMW-P53 protein. Taken together, our study provides critical insights into effective sensitization of OS cells through regulation of the cellular homeostatic machinery.

## Materials and methods

### Chemicals and reagents

Verteporfin (#SML0534-5MG), 2′,7′-dichlorofluorescin diacetate (DCFDA, #D6883), monodansylcadaverine (MDC, # D4008), Chloroquine (CQ, #C6528), and propidium iodide (PI; #P4864) were purchased from Sigma;. N-acetyl- L-cysteine (NAC, #47,866) and 3-[4, 5-dimethylthiazol-2-yl]-2,5-di-phenyltetrazolium bromide (MTT, #33,611) were obtained from SRL. LysoTracker Red DND-99 (LTR, # L7528), Enhanced Chemiluminescence (ECL, #32,106) and Antifade mountant (4ʹ-6-diamidino-2-phenylindole, #P36962) were procured from Thermo Fisher Scientific. Lipofectamine 3000 was from Invitrogen (#L3000-001). Primary antibodies were obtained from Santa Cruz Biotechnology (anti-β-actin #sc-69879, anti- GAPDH #sc-365062, anti-p53 #DO-1 sc-126 and anti-HSC70 #B-6 sc-7298); or Abcam (anti-LAMP-2A #Ab18528); or Bio Bharti Life Sciences (anti-p62 #BB-AB0130, and anti-HSP70 #BB-AB0210); or Cell Signaling Technology (anti-LC3B-II #D-11 3868S, anti-ATG-5 #D5F5U 12,994, anti-Rab-7 #D95F2 9367, anti-LAMP-1 #D2D11 9091S, anti-TFEB #D2O7D 37785S, anti-Beclin-1 #D40C5 3495 and anti-Caspase-3 #D3R6Y 14,220); and secondary antibodies- (anti-mouse #7076S and anti-rabbit #7074P2) were from Cell Signaling Technology. Plasmid GFP-RFP-LC3 was kindly provided by Dr. Sovan Sarkar (Birmingham Fellow, University of Birmingham). Ub GFP (#11,928) and mRFPUb (#11,935) plasmids were procured from Addgene. pCMV-Neo-Bam-p53-WT (Addgene Plasmid #16,434), pCMV-Neo-Bam-p53-R273H (Addgene Plasmid #16,439), and pCMV-Neo-Bam Empty Vector (Addgene Plasmid # 16,440) were a gift from Bert Vogelstein.

### In silico analysis

The transcriptomic data of OS patients were extracted from the GEO database of NCBI, which can be accessed through GEO accession ID GSE99671 [19]. Differentially expressed transcripts between the OS tumour sample and their tissue-matched control were identified using DESeq2 software [20]. The transcripts with p-value ≤ 0.05 were considered significantly differentially expressed. The resultant transcripts were then imported to CytoscapeV3.7.2 for highlighting the regulatory network consisting of differentially expressed transcripts. A Cytoscape plugin, ClueGO, was used to integrate Gene Ontology (GO) terms and KEGG pathways, which created a functionally organized GO/pathway term network [21].

### Cell culture

Human osteosarcoma cells- HOS (R156P mutant P53), breast cancer cells-MCF-7 (with WT P53) and MDA-MB-468 (with R273H-mutant-P53) cells were procured from NCCS (Pune, India); the non-small cell lung carcinoma cell line, H1299 (P53 Null) was a kind gift from Dr. Sanjeev Das (NII, New Delhi, India). Stable transfected P53 (R273H mutant P53 and WT P53) cell lines were made as described earlier [18]. Cells were cultured in 5% CO_2,_ at 37 °C, in medium supplemented with 10% fetal bovine serum (FBS) and antibiotics (1% penicillin–streptomycin).

### MTT assay

HOS cells were cultured in 96-well plates. Cells at 70–80% confluency were treated with specific compounds for time periods indicated in the figure. Following that, cytotoxicity was analyzed using MTT. Formazan crystals formed by live cells were dissolved in DMSO, and readings were captured at 570 nm with a differential filter of 630 nm by Multiskan GO microplate spectrophotometer [18]. Untreated samples served as control. The percentage viability in treated samples is relative to control of each time point.

### Drug uptake analysis

After treatment with VP, cells were harvested at 1800 rpm, 8 min. To the pellet, 500 μL PBS was added and centrifuged at 3000 rpm for 5 min. An equal volume of 100% acetonitrile was added, and cells were kept at vortex at medium speed for 30 min. This suspension was then centrifuged at 5000 rpm at RT for 10 min. The supernatant was collected in fresh eppendorf and 100 μL of this was added to wells of 96 well plate and absorbance was taken at 436 nm in Multiscan plate reader. A standard curve was plotted by taking the absorbance of the drug directly and then uptake in cells was analyzed using the equation.

### Measurement of caspase-3 activity

Approximately 2.5 × 10^5^ cells were seeded in a 6-well plate for estimation of caspase activity through caspase-3 colorimetric protease assay kit (Invitrogen). Briefly, proteins were extracted using RIPA buffer; Bradford assay was used to measure protein concentration and then the equal amount (60 μg) of protein was added to microtiter plates with a caspase-3 substrate (Ac-DEVD-pNA) and thereafter absorbance was measured at 405 nm in Multiscan plate reader.

### RNA isolation and cDNA preparation

TRIzol reagent (Invitrogen) was used to isolate total cellular RNA and cDNA was synthesized using GeneSure First Strand cDNA synthesis kit (Genetix) with random hexamers as per the manufacturer's instructions. cDNA templates were amplified for specific genes in CFX Connect Real-time PCR System (BioRad) and detected using SYBR Green (BioRad). GAPDH was amplified as a control. The relative RNA expression was calculated using Pfaffl's method.

### Transient transfection of plasmids

Briefly, cells were seeded at a density of 70%. The next day, cells were transfected with specific plasmid vectors (2 μg) using Lipofectamine 3000 reagent (Invitrogen, USA), as per the manufacturer's instructions.

### Immunoblotting

Western blotting was performed following methods described elsewhere [18]. Briefly, RIPA buffer (Sigma-Aldrich) was used for the extraction of proteins, and Bradford reagent for estimation. Proteins were loaded in lab-made denaturing polyacrylamide gels and transferred to polyvinylidene fluoride membranes (PVDF, Bio-Rad). Skimmed milk (HiMedia) was used for blocking. The blots were probed and re-probed with specific primary antibodies (dilution 1:1000) and detected using enhanced chemiluminescence (ECL; Thermo Fisher Scientific) on ChemiDoc (Bio-Rad). GAPDH (dilution 1:2000) was used as a loading control. Wherever required, the blots were cut to probe with different antibodies against proteins of different molecular weights. The make of antibodies is mentioned in the 'chemical and reagents' section.

### Monodansylcadaverine (MDC) staining

Cells (2.5 × 10^5^ cells/well) were seeded in a 6-well plate. After specific treatment, the cells were incubated in the dark at 37^∘^C for 15 min, with 0.05 mM MDC, and thereafter PBS wash was given and the cells were collected in 10 mM Tris–HCl containing 0.1% Triton X-10. Intracellular MDC fluorescence was measured by fluorescence photometry (excitation 380 nm and emission 525 nm) on a microplate reader (Fluoroskan Ascent).

### Estimation of intracellular reactive oxygen species (ROS)

Cells were seeded at a density of 8 × 10^3^ cells/well in 96-well plates and exposed to treatments. The ROS scavenger- NAC (20 mM) was added 1 h before the treatments. The cells were then washed in PBS, incubated with DCFDA (10 μM) for 30 min. Fluorescence was measured using a microplate reader (Fluoroskan Ascent) at 485 nm excitation and 530 nm emission.

### Lysotracker (LTR) staining

Cells were allowed to grow on a coverslip till 70% confluency and then loaded with VP. After incubation, the medium was replenished with a pre-warmed (37 °C) LTR-containing medium. The cells were then incubated for 20 min, solution aspirated, and slides were prepared. The cells were then observed under a fluorescence microscope (Zeiss, Axio Scope A1) and LTR fluorescence was compared between treated and control.

### Propidium iodide (PI) staining

Cells were seeded in 6 cm dishes at a density of 5 × 10^5^ cells/dish. The following day, the cells were treated with the desired drug for a required period of time. Thereafter, the cells were harvested, washed with PBS, and re-suspended in 500 μl of fresh PBS. To detect the percentage of dead cells, PI was added and incubated for 20 min in the dark. Thereafter flow cytometric (Cytoflex, Beckmann Coulter) analysis was performed and the acquired data were analyzed using CytExpert software.

### Acridine orange (AO) staining

For the determination of lysosomal permeabilization, cells were seeded in 6 well plate at a density of 2.5 × 10^5^ cells/well. The following day, cells were treated with the desired drug for a specific period of time. Thereafter, the cells were harvested, centrifuged for 10 min, washed with PBS, and re-suspended in fresh media. After that, AO was added (final concentration: 0.5 μg/mL), followed by incubation in the dark for 20 min. Cells were then washed with PBS and then re-suspended in fresh media. The samples were then acquired using a flow cytometer (Cytoflex, Beckmann Coulter) and analysis of acquired data was performed using CytExpert software. Percentage reduction in red fluorescence is represented through a bar diagram.

### Immunofluorescence microscopy

Cells were seeded on coverslips at 2.5 × 10^5^ cells/well and then treated with the drug as indicated. Cells were then washed with PBS and fixed with 100% methanol at − 20 °C for 10 min. After multiple PBS washes, blocking was done in 2.5% bovine serum albumin (BSA) for 60 min. The cells were incubated with primary antibody (1:500 dilution in 2.5% BSA) overnight at 4 °C, washed twice with PBS, and then incubated with FITC/TRITC conjugated secondary antibody (1:1000 in 2.5% BSA) for 60 min. Coverslips were mounted on slides using antifade DAPI and viewed under a fluorescent microscope (Zeiss, Axio Scope A1, or Axio Observer.Z1/7). Images were analyzed using Zen2.3 SP1 software.

### Statistical analysis

Data was analyzed using GraphPad Prism software version 5.0. The impact of specific treatment compared to control was statistically determined using one-way or two-way ANOVA. Multiple comparisons were analyzed using the Bonferroni method. If p-value was more than 0.05, then the difference was considered not significant (ns); whereas, if p-value was ≤ 0.05 it was considered significant and denoted by symbols * or # or $; if p-value ≤ 0.01 then denoted by ** / ## / $$ and if p-value ≤ 0.001 then denoted by *** / ### / $$$.

## Results

### Autophagy associated pathways are frequently dysregulated in OS patients

**Fig. 1:**
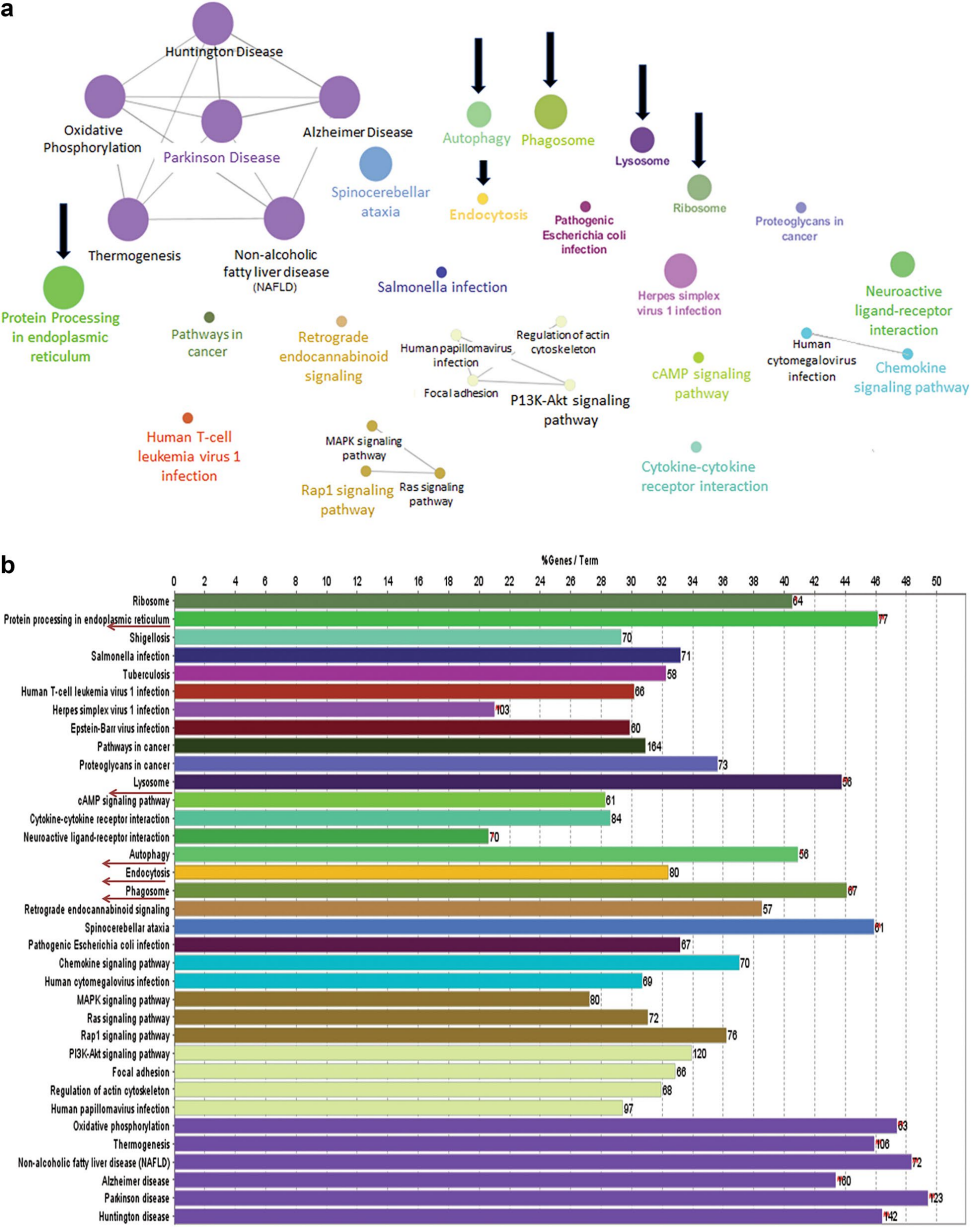
Autophagy associated pathways are dysregulated in OS. **a** ClueGo pathway map showing majorly dysregulated pathways and their connections in OS patients. **b** Bar graph representing the number of genes in each dysregulated pathway in OS patients

To understand the cause of the poor prognosis of OS, we analyzed the gene expression data of OS patients, extracted from GEO. Our objective was to identify the probable set of pathways that are maximally de-regulated and controlled by the significantly differentially expressed genes in patients suffering from OS. As evident from Fig. 1a, functionally linked pathways like cellular autophagy, phagosome, lysosome, protein processing and ribosome were amongst the significant deregulated pathways in patients suffering from OS. Figure 1a represents some of the top pathways found to be deregulated in OS, based on the gene expression data.

Furthermore, autophagy, endocytosis and phagosome were found to harbor a high percentage of differentially expressed genes implicating their probable involvement in the disease (Fig. 1b). Existing reports further validate the importance of autophagy signaling in OS promotion [22]. Additionally, several supportive studies are also there that reveal the pro-survival role of autophagy in cancer [6, 7, 23, 24]. The above evidence persuaded us to explore autophagy or disruption of protein homeostasis as a strategy to sensitize OS cells.

### VP sensitizes HOS cells

**Fig. 2:**
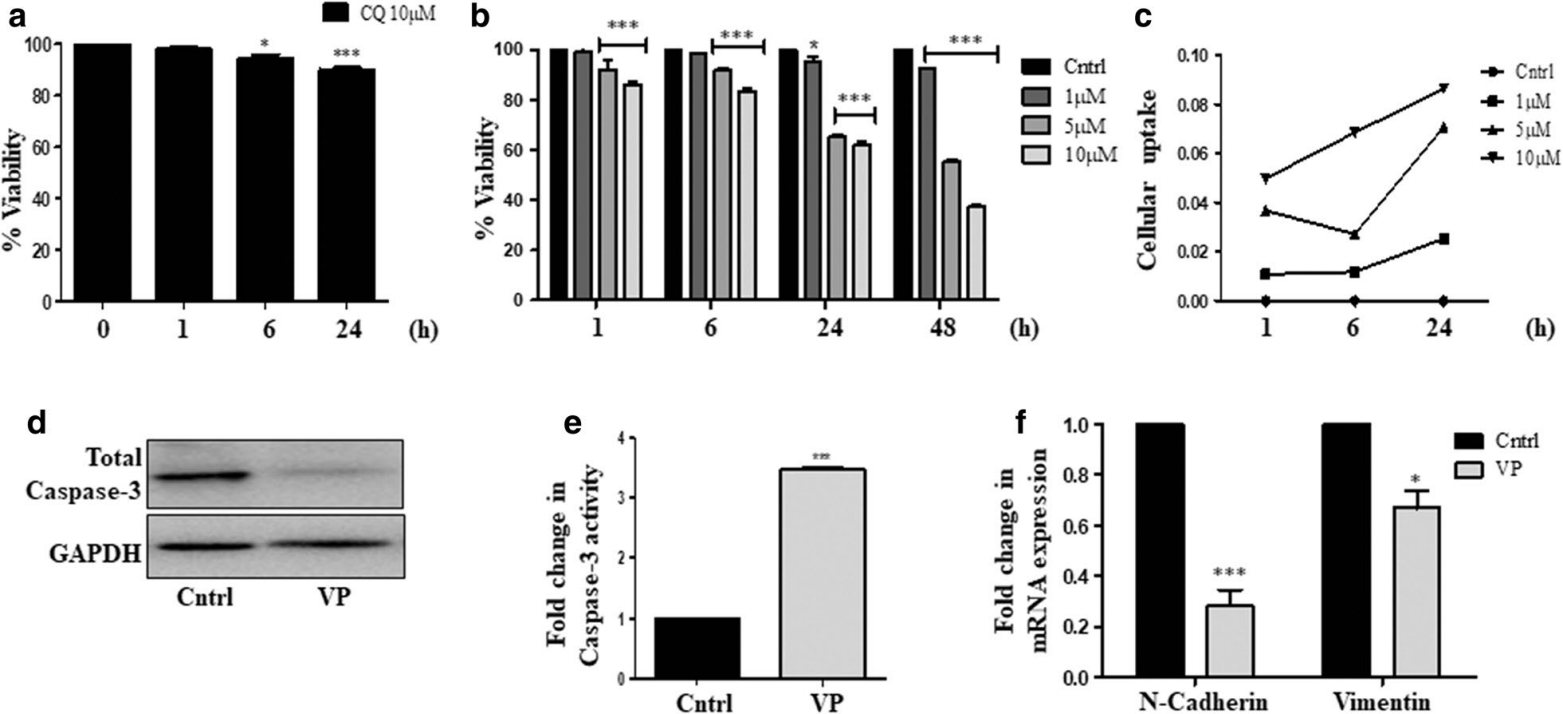
Effect of VP or CQ exposure on HOS cells. **a** Cell viability analysis, measured through MTT assay after exposure to CQ (10 µM). **b** Cell viability analysis, measured through MTT assay upon exposure to VP. **c** Cellular uptake after exposure to VP for different time points. **d** Immunoblot showing expression of total caspase-3 after VP exposure (10 µM) for 24 h. GAPDH is used as a loading control. **e** Fold change in caspase-3 enzyme activity after VP treatment (10 µM) for 24 h. Enzyme activity in untreated control is taken as '1′. **f** Gene expression of EMT markers was analyzed using RT-PCR after VP exposure (10 µM) for 24 h. Fold change is plotted taking gene expression of the untreated sample as '1′. ' ∗ ’ indicates a significant difference compared to untreated cells

We initially selected the well-established autophagy inhibitor and the FDA approved drug- chloroquine (CQ), which is known to inhibit autophagic flux by preventing autophagosome and lysosome fusion [25]. Cell viability was analyzed following CQ treatment for varied time points in human osteosarcoma cells (HOS). However, no cytotoxic effect was observed upon exposure to CQ at the specific doses (Fig. 2a). As CQ failed to show a significant cytotoxicity, we, therefore, opted for another FDA approved drug VP, known to possess potent light-independent autophagy inhibitory functions [26]. Cells were exposed to different doses of VP (1, 5 and 10 µM) for a varied time period (1, 6, 24 and 48 h) and cytotoxicity was analyzed. Interestingly, a dose and time-dependent cytotoxicity were observed with VP treatment (Fig. 2b). We further performed a VP uptake analysis in HOS cells. As depicted in Fig. 2c, a consistent increase in uptake of VP was observed over time. We thereafter chose a 10 µM dose of VP for a period of 24 h for experiments in the rest of our study. Further, the investigation into molecular mechanisms leading to VP-induced cytotoxic effect showed a decrease in expression of the total caspase-3 protein (Fig. 2d); an increase in caspase-3 activity (Fig. 2e) and a down-regulation of mesenchymal markers, like, N-cadherin and Vimentin after VP treatment (Fig. 2f).

### VP disrupts both early and late autophagic processes

**Fig. 3:**
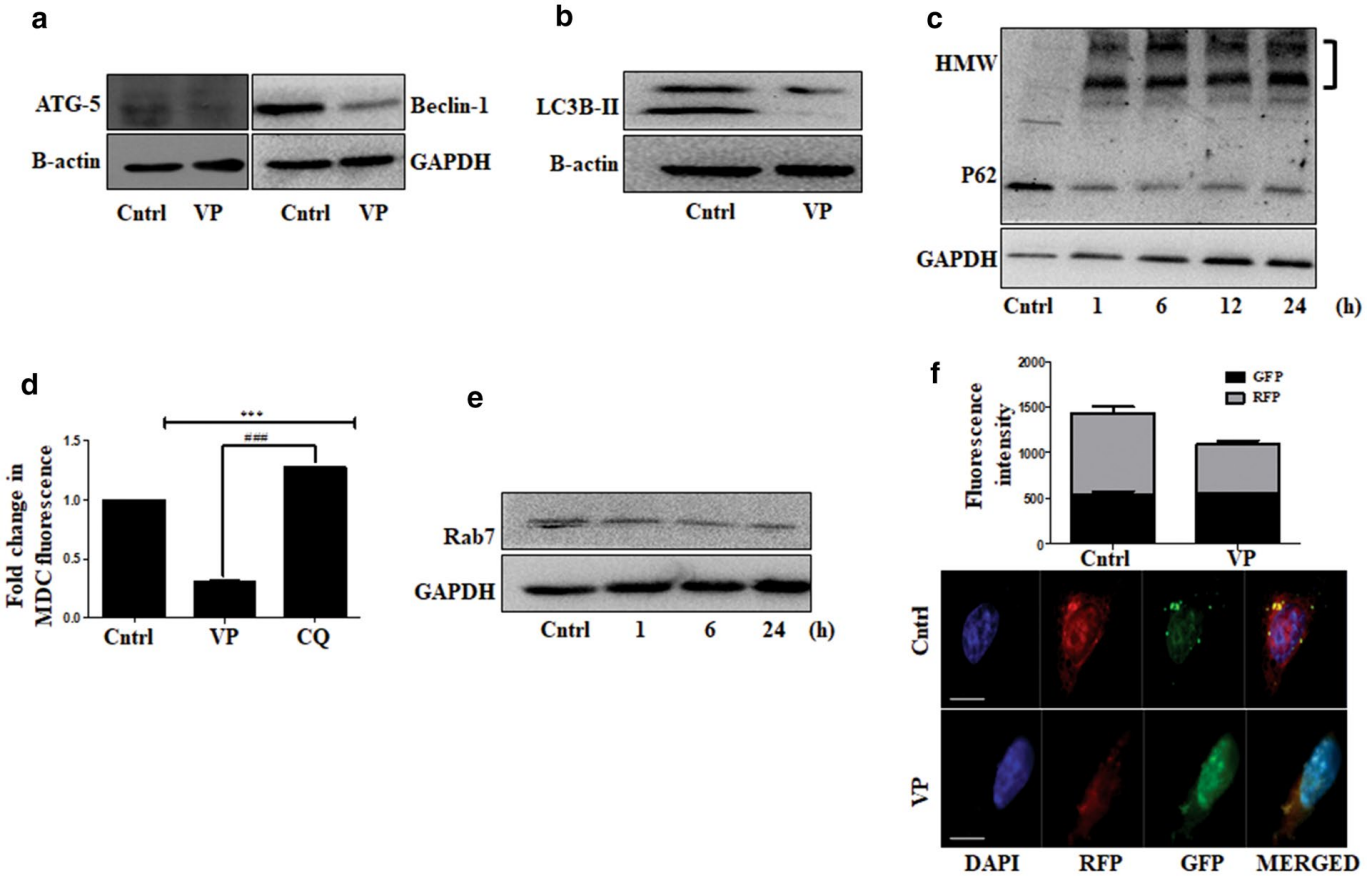
VP disrupts multiple steps of autophagy. **a**, **b** Immunoblots showing expression of ATG-5, Beclin-1 and LC3B-II after VP treatment for 24 h. **c** Immunoblot showing P62 protein expression after exposure to VP for different time points. The 'third bracket' represents the high molecular weight (HMW) band detected. **d** Fluorimetric analysis of MDC fluorescence in cells treated with VP or CQ (10 µM) for 24 h. **e** Immunoblot showing expression of Rab7 after VP exposure (10 µM). **f** GFP and RFP fluorescence intensity in cells transfected with GFP-RFP-LC3 vector followed by exposure to VP (10 µM) for 24 h. Scale bar: 10 µm. Objective: Plan Apochromat 63x/1.40 oil M27. * and # indicate the significant difference with respect to control and VP, respectively

We thereafter investigated whether VP has the potential to modulate the cellular homeostatic process autophagy in osteosarcoma cells. While VP is reported as an autophagy inhibitor, a detailed report exploring its effect on different steps involved in autophagy is least explored. VP-mediated inactivation of P62 protein is one of the most widely accepted effects on autophagy. Herein, we initially analyzed the expression of early autophagy markers required for the initiation of the autophagic process and extension of the phagophoric membranes. As evident in Fig. 3a, VP exposure showed a decrease in ATG-5 and Beclin-1 protein expression indicating disruption of early autophagic processes. We further analyzed the expression of LC3B-II. The level of lipidated form of LC3, popularly called as LC3-II, is commonly used to monitor the amount of autophagosomes present in a cell; LC3 is also required for autophagosome membrane expansion and fusion [27]. Importantly, the protein levels of LC3B-II decreased with VP treatment suggesting a probable reduction in autophagosome biogenesis after treatment (Fig. 3b). Furthermore, P62 showed the formation of a high molecular weight (HMW) band after VP treatment (Fig. 3c). P62 binds to LC3-II and facilitates autophagic cargo degradation [28]; a loss of P62 function is often associated with ubiquitinated protein accumulation, impaired autophagic trafficking and cell death [29]. Therefore a HMW-P62 band, as observed after VP exposure, reflects a compromised autophagy. An inhibition of autophagic vacuole formation after VP treatment was further confirmed using MDC staining. MDC is a fluorescent dye that preferentially accumulates in autophagic vacuoles [30]. The fluorimetric analysis revealed a decreased MDC fluorescence after VP exposure indicating a probable diminished number of cellular vesicles (Fig. 3d). On the contrary, CQ which is known to block autophagosome-lysosome fusion [25] showed an enhanced MDC fluorescence due to probable accumulation of vesicles, and hence served as a positive control. Further, Rab7 protein, known to have an important role in autophagic vacuole maturation [31], showed a drastic decrease in expression after VP treatment, suggesting that VP regulates expression of endosomal or autophagosomal trafficking markers as well (Fig. 3e). To further confirm that VP inhibits autophagy and reduces autophagosome-lysosome fusion, cells were transfected with the GFP-LC3-RFP vector, which helps to monitor autophagy [32]. Importantly, we didn't observe an increase in RFP fluorescence, indicating that VP inhibits autophagic flux (Fig. 3f). All the above suggests that the VP has a disruptive effect on the overall autophagic process in OS cells.

### VP disrupts lysosomal stability

**Fig. 4:**
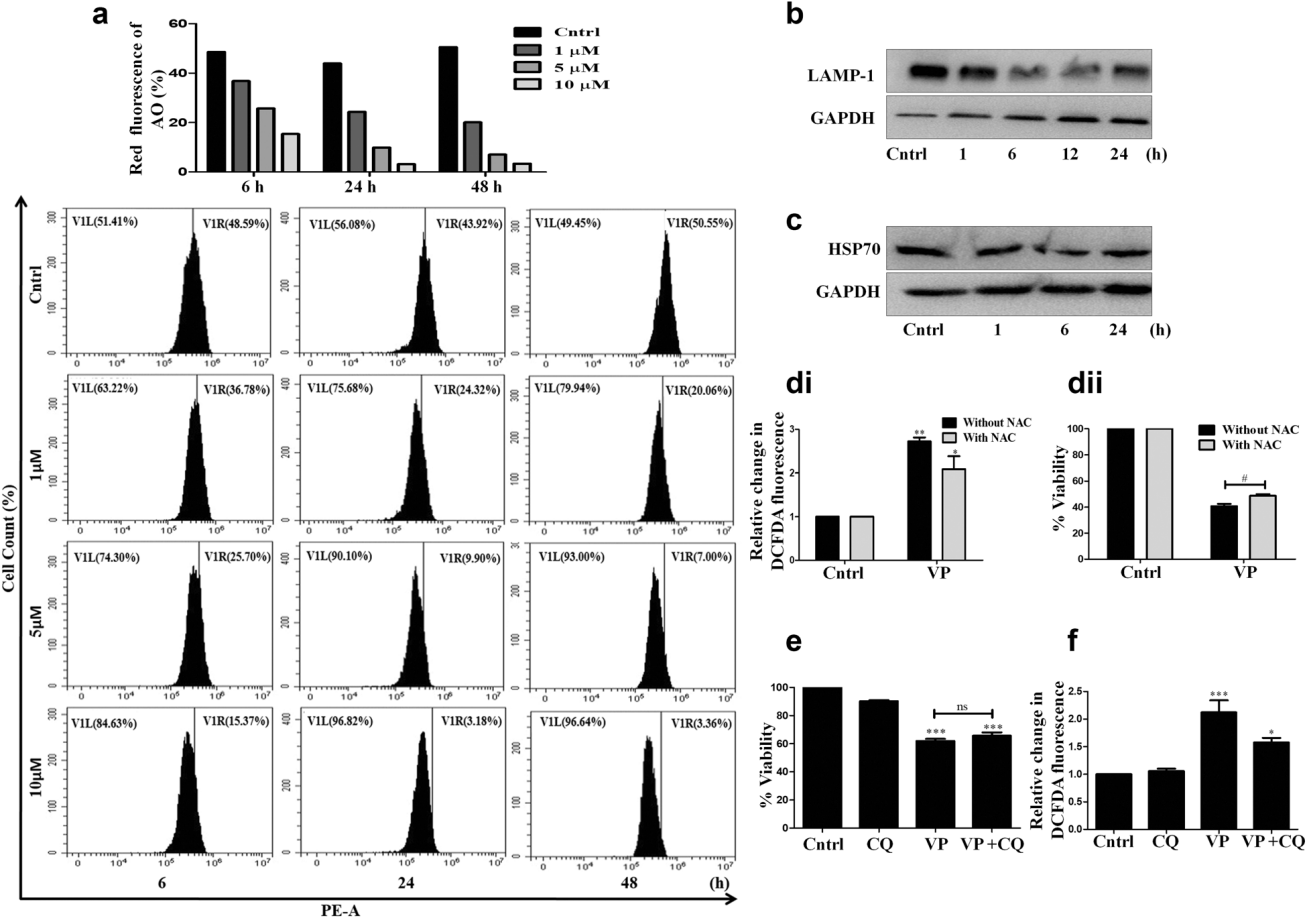
VP disrupts lysosomal stability. **a** Flow cytometric analysis representing a shift in AO fluorescence after VP treatment with respect to dose and time. **b**, **c** Immunoblots showing expression of LAMP-1 and HSP70 after VP exposure (10 µM). (**d**.i.) Bar graph representing fold change in intracellular ROS levels post-exposure to VP (10 µM) for 24 h as measured through DCFDA assay; (ii) MTT assay after 24 h of VP treatment, in presence or absence of NAC (20 mM). # indicates a significant difference compared to NAC treated cells. **e** MTT assay showing cell viability after treatment with CQ only (10 µM), or after VP (10 µM) plus CQ treatment for 24 h. CQ was added 1 h before VP treatment. **f** Fluorimetric analysis representing ROS levels in CQ only, or on VP plus CQ treatment. ‘*’ indicates a significant difference compared to untreated control

In the final step of autophagy, the cellular autophagic vesicles fuse with the lysosomes to degrade their contents. We observed that VP imparted a negative effect on the process of autophagy, however, its effect on the integrity of lysosomes was not known in this context. To analyze the same, we initially used the dye- acridine orange (AO), a lysosomotropic fluorochrome [33]. A flow cytometric analysis showed a decrease in red fluorescence after VP exposure, indicating disruption of lysosomal stability and probable lysosomal membrane permeabilization (LMP) (Fig. 4a). Lysosomal membrane proteins, such as LAMP-1, are also indicative of lysosomal integrity [34]. Interestingly, as evident from Fig. 4b, a decrease in LAMP-1 protein expression was observed after VP treatment. Heat-shock proteins like HSP70 are reported to act as an endogenous inhibitor of lysosome-mediated cell death [35]. We also observed a decrease in HSP70 expression upon VP exposure (Fig. 4c). Hydrolytic enzymes can leak out of lysosomes upon massive lysosomal breakdown, which in turn can lead to cellular stress [36]. Importantly, herein, a perturbation of lysosomal function was also associated with a noticeable increase in ROS, as measured through DCFDA assay (Fig. 4d.i). Pre-treatment of cells with the ROS scavenger- N-acetyl cysteine (NAC), resulted in a substantial decrease in VP-induced cytotoxicity, suggesting a positive correlation between enhanced ROS and cell death (Fig. 4d.ii). The assimilation of all the above results proposes VP to be a prototypical agent, with cytotoxic potential against OS, through inhibition of multiple steps involved in autophagy, including lysosomal de-stability; hence, its action is not just restricted to P62-HMW complex formation.

CQ has often been found to enhance the cytotoxic effects of many chemotherapeutic agents [37]. It is known to inhibit the autophagosome-lysosome fusion process [25] leading to enhanced accumulation of vesicles, which often triggers cell death. Hence, we assumed that CQ might have an additive effect with VP and can be considered in combination for augmented benefit against OS. However, as shown in Fig. 4e, CQ and VP co-treatment showed no cumulative cytotoxic effect. Additionally, CQ either alone or in combination with VP had minimal effect on intracellular ROS levels at the dose studied further suggesting the ineffectiveness of this combination (Fig. 4f). We assume that, since both the compounds inhibit autophagic flux, they did not manifest an additive effect. Also, the fact that only CQ exposure, at the dose studied, did not impart a significant cytotoxicity in the HOS cells might have contributed to the observed ineffectual effect of the combination. We further checked for the difference in expression of the autophagic markers like, LC3B-II and P62 with VP plus CQ treatment, and in corroboration to the above results it failed to show a significant difference in expression compared to independent treatments (Additional file 1: Fig. S1a.i. and a.ii).

### Proteasomal inhibitor enhances VP-induced cytotoxicity

**Fig. 5:**
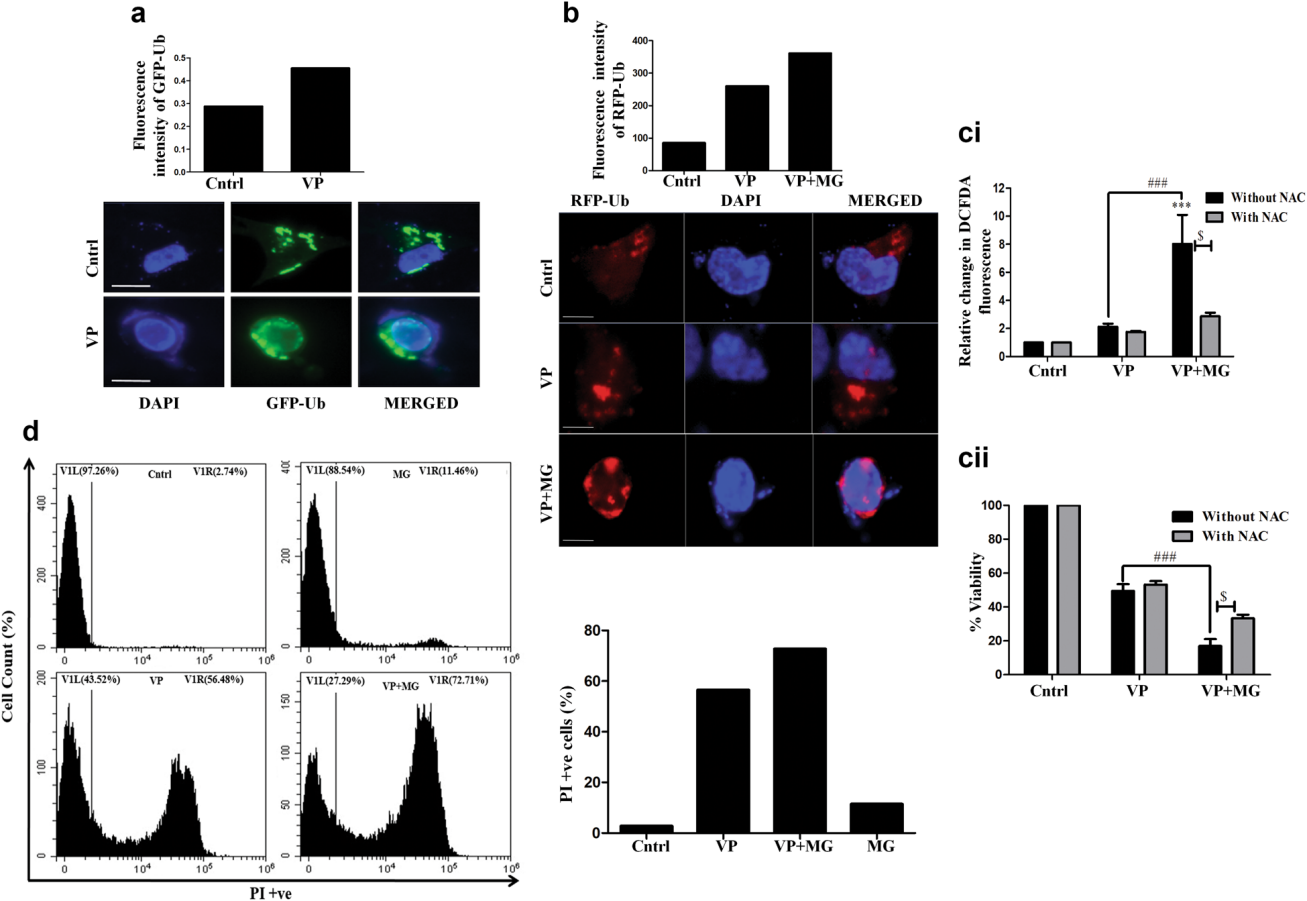
MG co-treatment enhances cytotoxicity. **a** Fluorescence microscopy showing GFP-Ub after VP exposure (10 µM) for 24 h. Scale bar: 100 µm. **b** Fluorescence microscopy showing RFP-Ub upon VP (10 µM) only, or VP and MG (0.5 µM) treatment for 24 h. **c** (i) Bar graph representing fold change in intracellular ROS levels post-exposure to VP only, or VP plus MG for 24 h. (ii) MTT assay analyzing cell viability after 24 h of VP or VP plus MG treatment in the presence or absence of NAC. *, # and $ indicate the significant difference with respect to control, VP with VP + MG, or between NAC^+^ and NAC^−^ cells, respectively. **d** Flow cytometric analysis showing PI^+ve^ cells after VP (10 µM) and/or VP plus MG (0.5 µM) treatment for 24 h

While we were in search of a molecule that can enhance VP-induced effects, we came across existing literature that points towards the functional crosstalk existing between the cellular ubiquitin proteasomal degradation pathway (UPS) and autophagy that together maintains cellular proteostasis [38]. In this regard, autophagy impairment is known to hinder proteasomal substrate delivery due to excess accumulation of p62, thus compromising UPS function [28, 29]. This allowed us to apprehend that a simultaneous blocking of UPS alongside VP can possibly further enhance VP-induced effects. Explicitly, with autophagy dysregulated and P62 forming HMW protein aggregate, the addition of a proteasomal inhibitor might further disrupt protein clearance leading to increased cytotoxicity. To explore a probable perturbation of protein homeostasis after VP exposure, we analyzed the formation of GFP-Ub punctaes after VP treatment. As evident from Fig. 5a, an increase in green punctate dots was visible with VP exposure indicating Ub protein accumulation. We thereafter exposed cells to the proteasomal inhibitor- MG132 along with VP and analyzed RFP-Ub fluorescence microscopically. As expected, enhanced accumulation of RFP-Ub puncta was observed in cells treated with VP and MG compared to only VP exposure (Fig. 5b) showing an additive accumulation of Ub proteins. Additionally, ROS levels showed a significant elevation (Fig. 5c.i) along with a correlative increase in cytotoxicity in the combination treatment, as measured through MTT assay (Fig. 5c.ii). Importantly, NAC reduced the cytotoxic effect suggesting a role of ROS in imparting the cytotoxic response in osteosarcoma cells (Fig. 5c.ii). A similar cytotoxic effect was also observed when lung cancer cells- H1299 were exposed to VP and MG (Additional file 1: Fig. S1b, c and d). An enhanced ROS and associated decreased cell viability were observed in the H1299 cells with different P53 protein status (Additional file 1: Fig. S1b, c and d). Application of another proteasomal inhibitor- ALLN in combination with VP (Additional file 1: Fig. S1e) also showed enhanced cytotoxic effect in HOS cells, compared to only VP or ALLN. To further confirm enhanced cytotoxicity with MG and VP treatment, we also measured the percentage of PI^+ve^ cells after exposure to both the compounds. The PI dye is impermeable to live cells but stains dead cells; therefore, an increase in PI^+ve^ cells, as observed, confirmed more cell death after combination treatment (Fig. 5d). From the above experiments, it can be inferred that a proteasomal inhibitor increases VP-induced cellular stress leading to enhanced cell death.

### VP induces ROS-dependent HMW P53 band

**Fig. 6:**
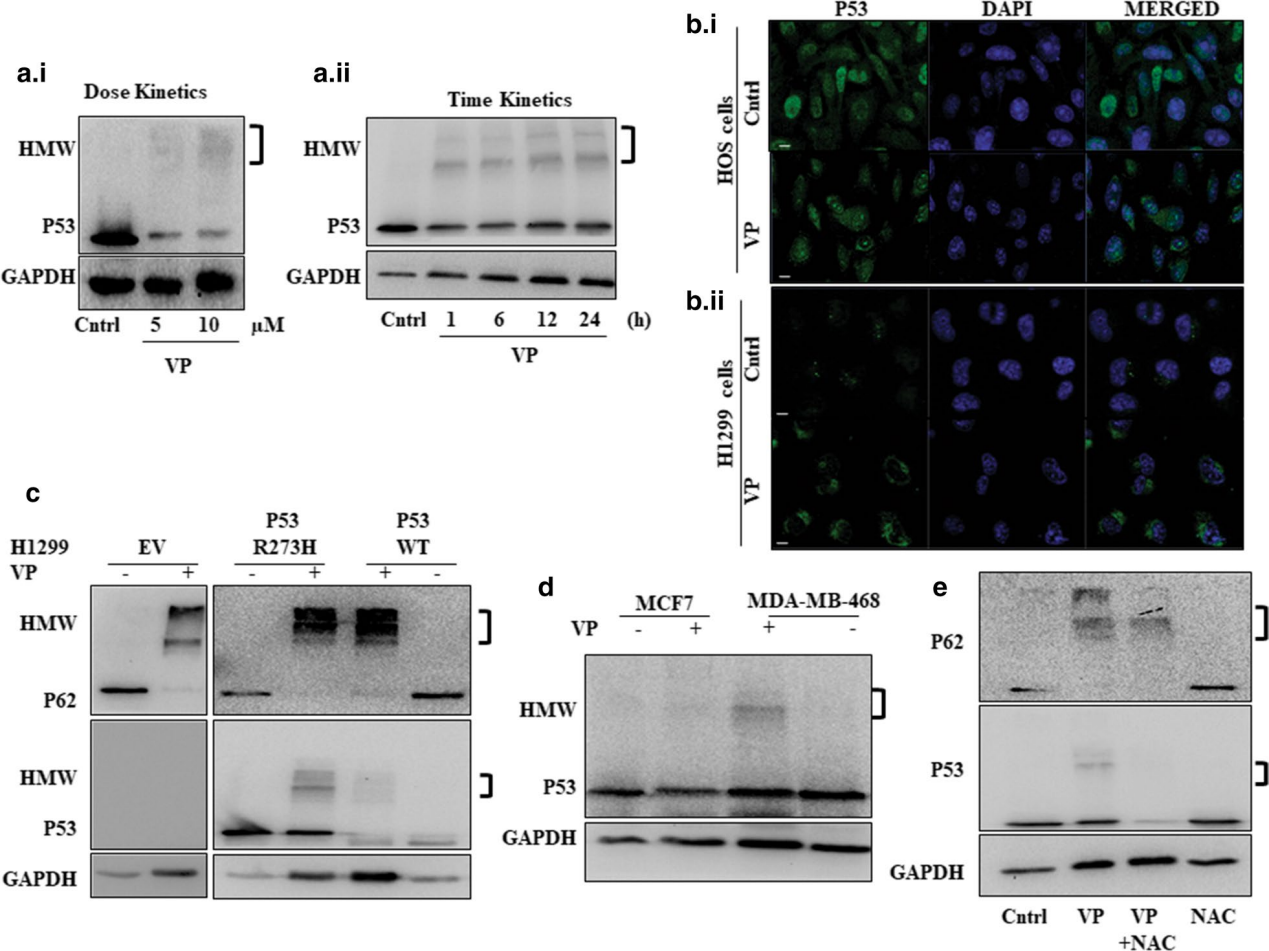
VP induces HMW protein of P53. Immunoblots showing P53 expression after VP treatment (10 µM) at different doses (**a**.i.), or (**a**.ii.) and different time points in HOS cells. (**b**.i) Immunofluorescence images showing P53 protein upon VP exposure in HOS, and (**b**.ii) H1299 cells stably transfected with GOF-R273H-P53 cells. Scale bar: 10 μm. Objective: Plan Apochromat 63x/1.40 oil M27. **c** Immunoblots showing P62 and P53 expression upon VP treatment in H1299 cells stable transfected with EV or GOF-R273H-P53 or WT-P53. **d** Immunoblots showing P53 expression after VP treatment in MCF-7 and MDAMB-468 cells. **e** Immunoblots showing P62 and P53 expression upon VP treatment with or without NAC in HOS cells. The ‘third bracket’ indicates the high molecular weight (HMW) band detected

As discussed earlier, mutations in P53 play a pivotal role in OS development and therapy. We were therefore interested in investigating whether VP regulates stability of the P53 protein. Interestingly, as shown in Fig. 6a.i and a.ii., similar to what was observed for P62 protein, VP exposure at different doses or escalating time showed a HMW band in HOS cells when probed for P53 by immunoblot. To confirm the same, we performed immuno-fluorescence analysis of P53 protein in the HOS cells (harbouring a GOF-mutant-P53) (Fig. 6b.i) [39] and also in H1299 null cells stably transfected with GOF-P53. Predominantly, cytoplasmic accumulation of green fluorescence of P53 protein was observed in both the cell types, more distinctly in the stable transfected p53 overexpressing cells (Fig. 6b.ii). Thereafter, we analyzed HMW aggregate formation in the H1299 cells stably transfected with either empty vector (EV), or WT-P53, or GOF-R273H-P53 after VP exposure. As expected, no HMW band was observed in the EV transfected cells, but importantly, cells stably transfected with wild type or GOF-R273H-P53 showed a HMW band when probed for P53 (Fig. 6c); however, the HMW band intensity was more for the GOF cell type, though HMW-P62 did not follow a similar trend. Interestingly, upon pull-down of P53 protein in VP-treated cells, P62 was not found to be co-immuno-precipitated, specifying that P53 and P62 proteins were not part of the same HMW complex in this study (data not shown). The HMW-protein band for P53 was also confirmed in two other cell types, MCF7 (WT-P53) and MDA-MB-468 (GOF-R273H-P53) (Fig. 6d) [40]. Earlier studies depict that protein oxidation by ROS can play a significant role in formation of high molecular weight protein aggregates [41]. Therefore, to further understand whether generation of ROS has a role in VP-induced formation of HMW protein aggregates, we treated the cells with the ROS quencher- NAC. As evident from Fig. 6e, a decrease in both VP-induced P62 and P53 HMW band was observed with the addition of NAC, suggesting that ROS plays a critical role in HMW complex formation.

### MG co-treatment induces lysosomal targeting of selected proteins

**Fig. 7:**
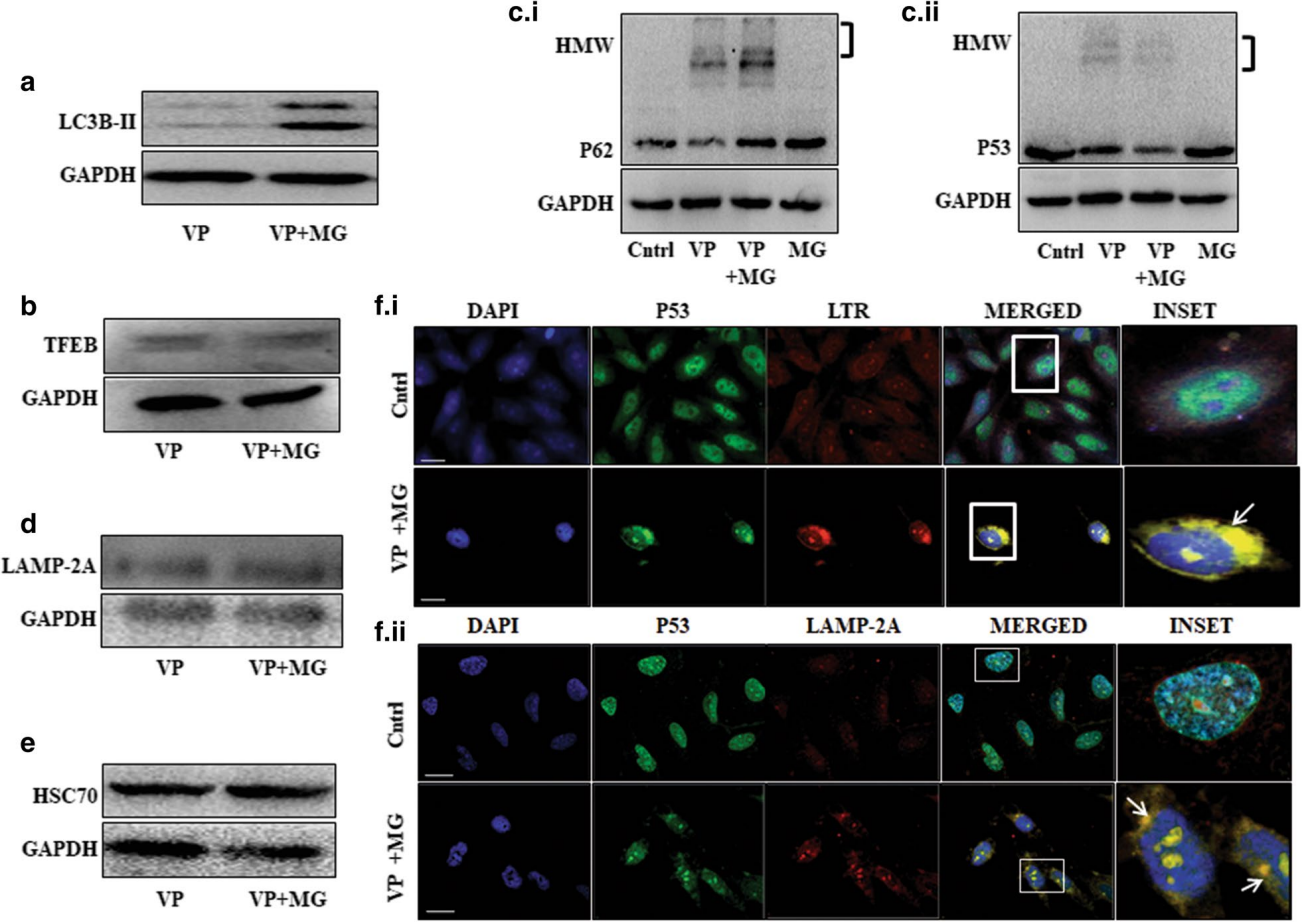
VP plus MG co-treatment targets P53 to lysosomes. Immunoblots showing LC3B-II (**a**), TFEB (**b**), P62 (**c**.i) and P53 (**c**.ii.) expressions after VP (10 µM) or VP plus MG treatment for 24 h. The ‘third bracket’ represents an HMW band detected. **d** and **e** Immunoblots showing LAMP-2A and HSC70 expression after VP or VP plus MG treatment. (**f**.i) Immunofluorescence image showing P53 (FITC-green) and Lysotracker-Red; (**f**.ii) or P53 (FITC-green) and LAMP-2A (TRITC-red) post VP and MG treatment. White arrow indicates co-localization. Scale bar: 10 μm. Objective: Plan Apochromat 63x/1.40 oil M27

There are existing studies that hint of an increase in the autophagic process after proteasomal inhibition [18, 42]. On a similar note, an increased LC3B-II protein level was observed in MG plus VP treated cells compared to only VP (Fig. 7a). Simultaneously, an increase in red fluorescence of AO (Additional file 1: Fig. S1f) and TFEB protein expression was also seen in the combination treatment (Fig. 7b); TFEB is reported to be involved in lysosomal biogenesis [43]. Collectively, these observations suggest that MG can partly salvage autophagy-like phenomena. Interestingly, as we further analyzed P62 and P53 protein expression in the combination treatment, we observed an intense HMW-P62 band in the presence of both MG and VP, compared to only VP. However, in contrast, the P53-HMW protein did not show an increase in the combination treatment (Fig. 7c.i and ii). Kaushik et al. in 2008, earlier reported that impairment of macroautophagy could lead to an accompanying activation of a special branch of autophagy, like, the chaperone-mediated autophagy (CMA) that can contribute to selective protein degradation [44]. Also, a recent study suggests that reduced macroautophagy can activate CMA leading to clearance of p53 [45]. During CMA, selected cytoplasmic proteins are recognized by chaperones and directed to lysosomes via lysosomal membrane-protein (LAMP-2A) [46]. As we analyzed protein expression, we found an increase in the expression of LAMP-2A in VP plus MG exposed cells (Fig. 7d). Additionally, HSC70- an important CMA marker that recognizes specific KFERQ-like sequence motifs on CMA substrates also showed an increased expression in the combination treatment (Fig. 7e). Importantly, P53 is reported to be a CMA substrate with a KFERQ motif [47]. Next, we analyzed co-localization of P53 with LTR; an increased co-localization of P53 with LTR was observed in VP plus MG-treated cells (Fig. 7f.i). We then probed for P53 protein and observed its co-localization with LAMP-2A through immune-staining which also followed a similar pattern (Fig. 7f.ii.). We hence propose that the combination of VP and MG targets P53, and not P62, to the lysosomes, possibly through a special branch of autophagy.

## Discussion

**Fig. 8:**
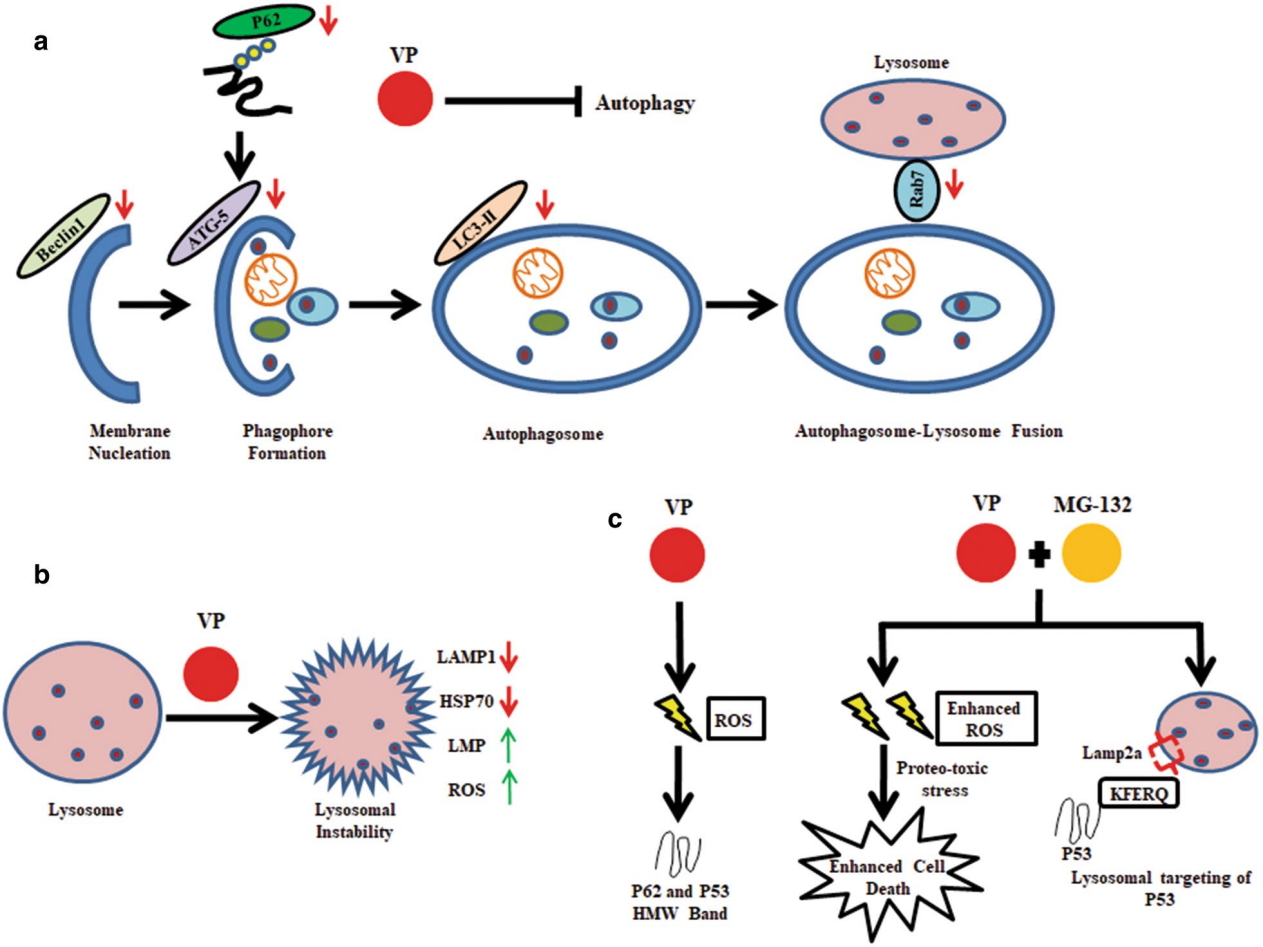
Schematic representation of VP induced effects. **a** and **b** Effects of VP on the autophagic pathway in HOS cells. Down (↓) pointing red arrow and up (↑) pointing green arrow represents a down-regulation or up-regulation respectively after VP treatment. **c** The overall effect of VP and MG combination on HOS cells

Autophagy is known to enhance the survival fitness of tumor cells [48]. The inhibition of autophagy by the use of pharmacological inhibitors is reported to increase the sensitivity of drugs in multiple cancer types [49, 50]. Given the significant implication of autophagy in cancer, including OS, we planned to target autophagy or cellular protein homeostasis machinery as a therapeutic strategy. While exploring for FDA approved drugs that possess autophagy modulating functions, we came across VP, a widely accepted photosensitizer [26]. According to NCI's information on clinical trials, VP is re-purposed as a PDT drug for Phase II trials in multiple cancers. In addition, several reports portray VP to be a potent anti-cancer agent [51, 52]. Importantly, VP is known to inhibit autophagy independent of light [53]. Given that autophagy is highly dysregulated in osteosarcoma, in this study we explored the potential of VP as an effective drug against OS. A recent report also shows the use of VP in impairing the growth and migration of OS cells in vitro. VP was found to act through inhibition of YAP-TEAD association and ROCK2 protein function [54]. Herein, we show that VP inhibits both early steps of autophagy and disrupts late-stage autophagosome-lysosome fusion in OS cells. We observed that VP reduced the expression of proteins like ATG-5, Beclin-1, LC3B-II. It further induced P62 protein-HMW aggregate formation indicating a compromise in autophagic vesicle formation and cargo delivery as well. Figure 8a and b schematically represents the effect of VP on autophagy in OS cells. The impaired autophagic function was also associated with an increase in lysosomal permeabilization coupled to an enhanced ROS and increased ROS-dependent cytotoxicity. Figure 8b shows the effect of VP on lysosomes. Importantly, CQ an inhibitor of autophagosome-lysosome fusion, failed to impart a significant cytotoxic effect confirming that the cytotoxicity observed upon VP treatment cannot be exclusively attributed to its impact on autophagic flux only. Though there have been independent studies showing VP-induced protein oligomerization and lysosomal pH change [55], yet, a holistic study indicating its effects on the overall process of autophagy and its connection with cell cytotoxicity, especially in OS cells is missing.

Also, there is burgeoning evidence for connection between two major and evolutionary conserved degradation machineries: autophagy and UPS. Several studies show the compensatory relation between the two pathways [56]. Inhibition of UPS using various proteasomal inhibitors is known to up-regulate autophagy [57]. Similarly, impairment of autophagy is reported to be correlated with both activations of UPS or delayed cargo delivery to UPS due to P62 accumulation [58]. In this regard, we observed that VP exposure resulted in a significant accumulation of ubiquitinated proteins coupled to P62 accumulation and associated autophagy inhibition. These cellular protein aggregates are generally cleared off by the UPS, thus mitigating proteo-toxic stress [59]. With autophagy inhibited by VP, we explored whether inhibition of the other intracellular protein turnover machinery, the UPS can aggravate cytotoxicity. Increased cytotoxicity accompanied by enhanced ROS was observed with VP plus MG not only in osteosarcoma cells studied but also in lung cancer cells with different P53 protein status, suggesting that it might not be a cell type dependent event.

Previous studies report cross-linked HMW complex formation with VP, but predominantly for P62 [41, 60]. Herein, we show that upon VP exposure, a HMW band is also observed when lysates are probed with anti-P53 antibody. A possible explanation for a P62-HMW complex formation with VP is often credited to the presence of the PB1 (Phox and Bem1p-1) domain with a scaffold-like grasping fold that might facilitate its homo- and hetero-oligomerization [41]. But the P53 protein is not known to have a similar PB1 domain. It prompted us to explore any structural similarity existing between P53 and P62. However, amino acid alignment of P53 with P62 failed to reveal by far any particular shared sequence among them which might contribute to its HMW complex formation. But it is worthwhile to mention here that, P53 protein is reported to be naturally 'sticky' and to undergo multiple oligomeric states and higher-order aggregates by virtue of its oligomerization domain [61]. Further, mutation in P53 protein can make them even more susceptible to oligomerization as it can facilitate interaction with other stabilizing proteins thus increasing their overall stability [62]. Here, we assume that VP most likely stimulates this already existing oligomerization potential of P53 and its mutants; however, precise mechanisms involved in such phenomenon are required to be further explored. Importantly, we identified that the average hydropathicity score of P62 is about 17% lower than that of P53, indicating that P62 protein is more hydrophobic than P53. Hence, P62 is more likely to show an enhanced oligomerized state than P53 [63]. Mutant P53 is further reported to misfold and co-aggregate with its paralogs, like, P63 and P73 and/or other proteins as well [64]. But here, P53 was not found to be part of the P62-HMW aggregate, indicating that it was independent of P62. However, the HMW bands for both the proteins reduced with NAC suggesting a role of ROS in its formation, independent of the type of proteins oligomerized. In corroboration to above, Donohue et al. in 2014 showed HMW-P62 crosslinks to be formed resultant of ROS, especially singlet oxygen (^1^O_2_) generated after VP treatment [41]. Further, the P62-HMW protein band, but not P53 enhanced with the addition of MG. Proteasomal inhibitors are known to enhance a selective branch of autophagy [65]; also, P53 is a probable substrate for CMA [45, 47]. In corroboration to above, we observed that P53 co-localized with LTR and also with LAMP-2A upon VP plus MG exposure indicating a probable targeting to lysosomes. Images of all original data are available in Additional file 2. Figure 8c shows a schematic representation of effect of VP and MG.

## Conclusion

To the best of our knowledge, we are the first to report VP-induced sensitization of OS cells through inhibition of multiple steps of autophagy. We also propose that VP exposure results in HMW band formation for P53 protein. This has significant implications in cancer therapy as mutant P53 is highly prevalent in OS which can hence be targeted by VP. We also show that MG can enhance VP-mediated cytotoxicity in OS cells and targets P53 to lysosomes. Our results can have a deep therapeutic impact on OS therapy in future.

## Supplementary Information

**Additional file 1: Fig. S1.** (a.i.) or (a.ii.) Immunoblots showing expression of LC3B-II and P62 upon VP (10 µM) and/or CQ (10 µM) treatment for 24 h in HOS cells. (b, c and d) Bar graph representing fold change in intracellular ROS levels, and MTT assay analyzing cell viability after 24 h of VP or VP plus MG treatment in the presence or absence of NAC in H1299-GOF-R273H-P53 (b.i and b.ii), H1299-WT-P53 (c.i. and c.ii.) and H1299-EV (d.i. and d.ii.) cells. # indicates a significant difference with respect to VP. e. MTT assay showing cell viability after ALLN (10 µM) and/or VP (10 µM) treatment for 24 h in HOS cells. *, # represents a significant difference with respect to untreated control and VP treated cells, respectively. f. Flow cytometry images showing fluorescence of AO upon treatment with VP (10 µM) only or VP plus MG (0.5 µM) in HOS cells.

**Additional file 2.** Images of all original data.

## Data Availability

The datasets used and/or analyzed during the current study are available from the corresponding author on reasonable request.

## Abbreviations

OS: Osteosarcoma
CQ: Chloroquine
VP: Verteporfin
UPS: Ubiquitin proteasomal system
ROS: Reactive oxygen species
MDC: Monodansylcadaverine
LC3B-II: Microtubule-associated protein light chain 3-II
HMW: High molecular weight
PB1: PB1 (Phox and Bem1p-1)
DCFDA: 2′ 7′ –Dichlorofluorescin diacetate
AO: Acridine Orange
