# Supplementary figures and images for: Verteporfin disrupts multiple steps of autophagy and regulates p53 to sensitize osteosarcoma cells
Source: Cancer Cell Int. 2021 Jan 14;21:52. doi: 10.1186/s12935-020-01720-y (PMC7807844; doi:10.1186/s12935-020-01720-y)

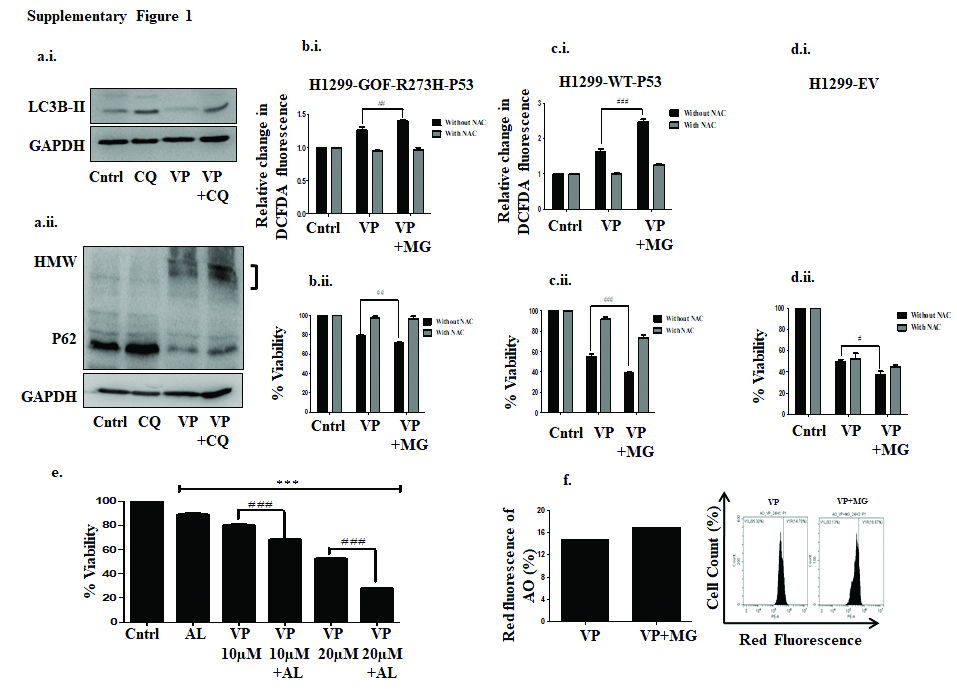

Supplement: Supplementary file 1 — Additional file 1: Fig. S1. (a.i.) or (a.ii.) Immunoblots showing expression of LC3B-II and P62 upon VP (10 µM) and/or CQ (10 µM) treatment for 24 h in HOS cells. (b, c and d) Bar graph representing fold change in intracellular ROS levels, and MTT assay analyzing cell viability after 24 h of VP or VP plus MG treatment in the presence or absence of NAC in H1299-GOF-R273H-P53 (b.i and b.ii), H1299-WT-P53 (c.i. and c.ii.) and H1299-EV (d.i. and d.ii.) cells. # indicates a significant difference with respect to VP. e. MTT assay showing cell viability after ALLN (10 µM) and/or VP (10 µM) treatment for 24 h in HOS cells. *, # represents a significant difference with respect to untreated control and VP treated cells, respectively. f. Flow cytometry images showing fluorescence of AO upon treatment with VP (10 µM) only or VP plus MG (0.5 µM) in HOS cells. [file 12935_2020_1720_MOESM1_ESM.tif]
