# Supplementary material for: Verteporfin disrupts multiple steps of autophagy and regulates p53 to sensitize osteosarcoma cells
Source: Cancer Cell Int. 2021 Jan 14;21:52. doi: 10.1186/s12935-020-01720-y (PMC7807844; doi:10.1186/s12935-020-01720-y)
Supplement: Supplementary file 2 — Additional file 2. Images of all original data. [file 12935_2020_1720_MOESM2_ESM.ppt]

## Slide 1
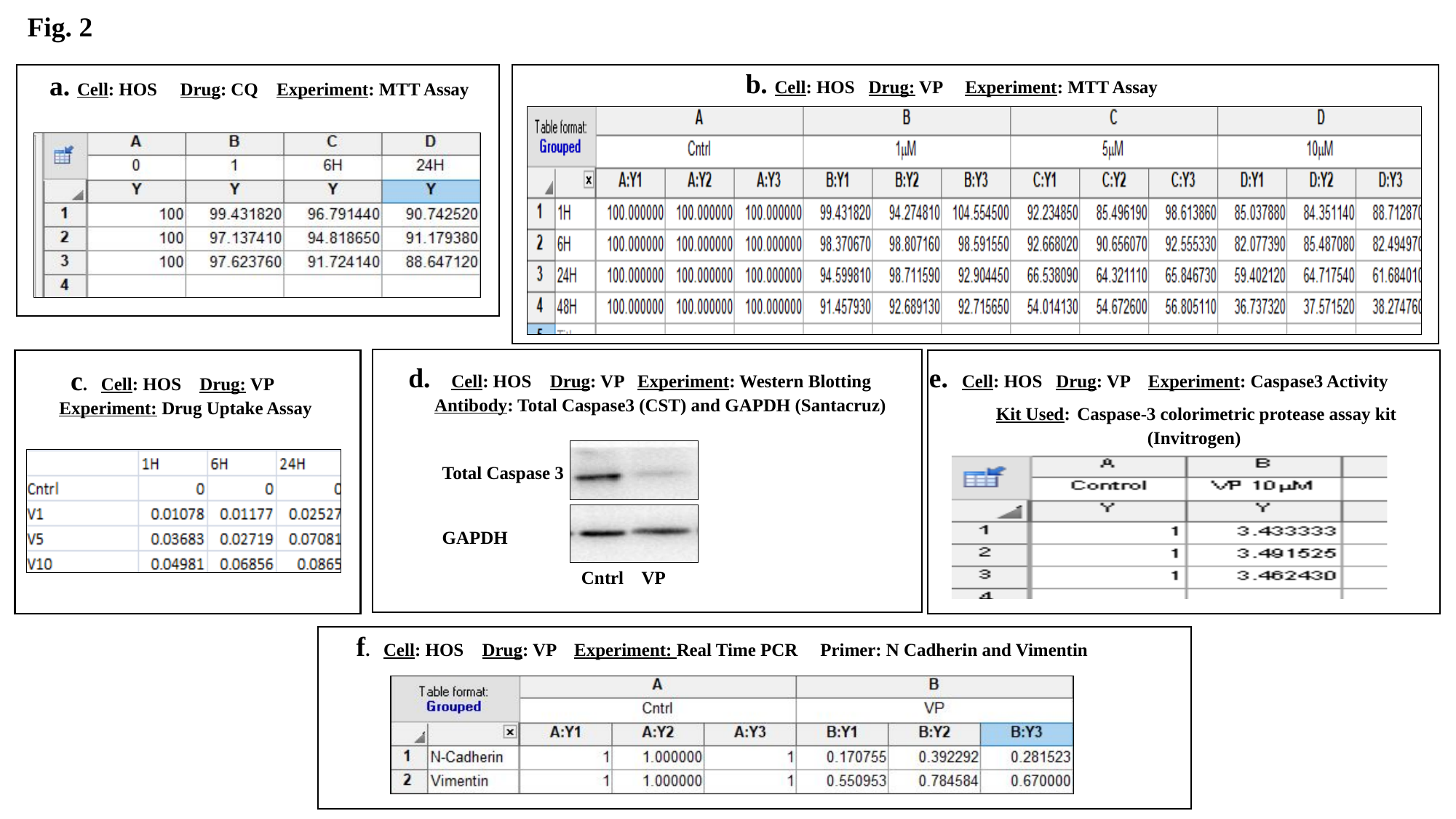

Fig. 2
b. Cell: HOS Drug: VP Experiment: MTT Assay
 a. Cell: HOS Drug: CQ Experiment: MTT Assay
d. Cell: HOS Drug: VP Experiment: Western Blotting Antibody: Total Caspase3 (CST) and GAPDH (Santacruz)
e. Cell: HOS Drug: VP Experiment: Caspase3 Activity Kit Used: Caspase-3 colorimetric protease assay kit (Invitrogen)
c. Cell: HOS Drug: VP Experiment: Drug Uptake Assay
Total Caspase 3
GAPDH
Cntrl VP
f. Cell: HOS Drug: VP Experiment: Real Time PCR Primer: N Cadherin and Vimentin

## Slide 2
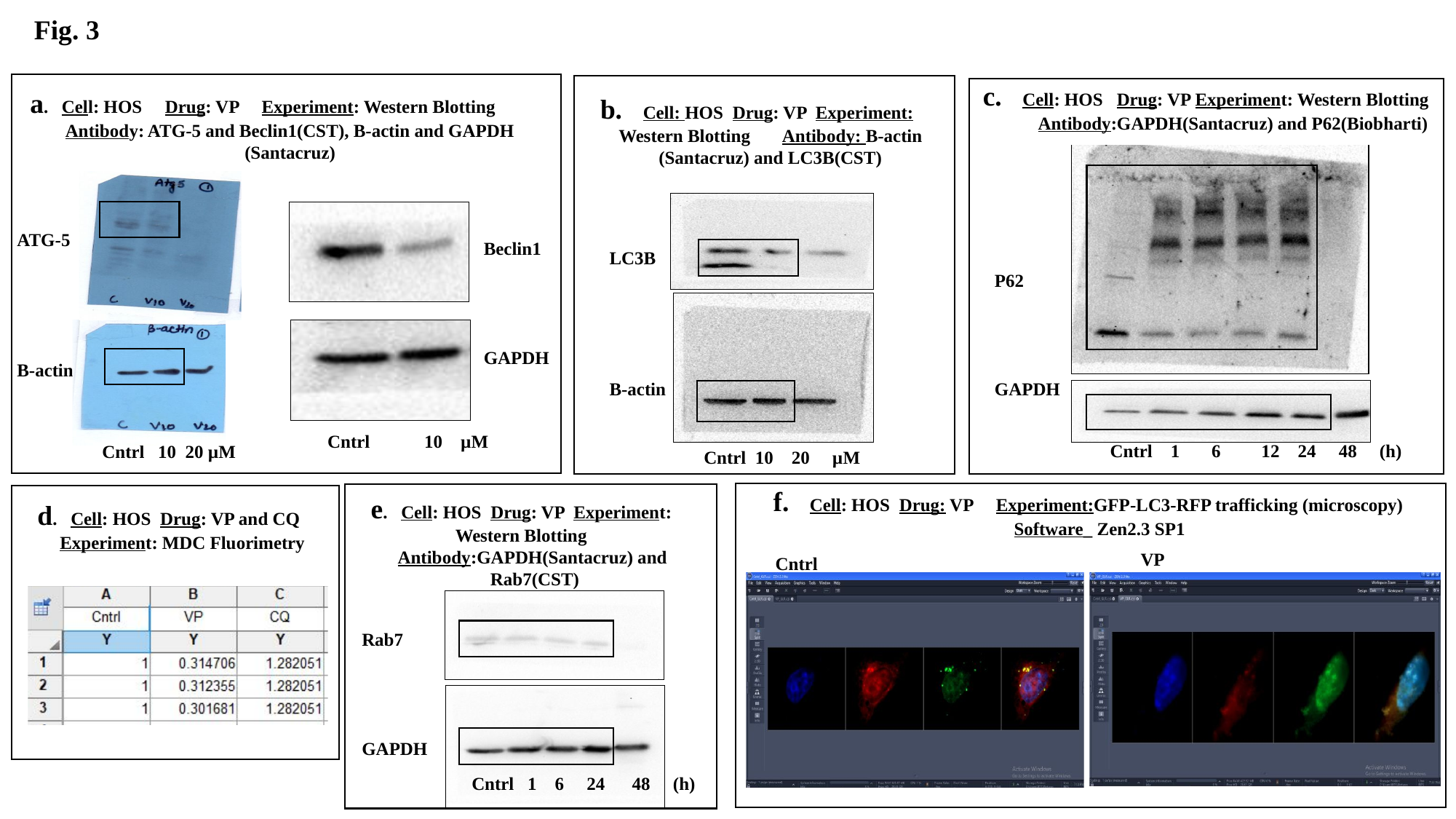

Fig. 3
c. Cell: HOS Drug: VP Experiment: Western Blotting Antibody:GAPDH(Santacruz) and P62(Biobharti)
a. Cell: HOS Drug: VP Experiment: Western Blotting Antibody: ATG-5 and Beclin1(CST), B-actin and GAPDH (Santacruz)
b. Cell: HOS Drug: VP Experiment: Western Blotting Antibody: B-actin (Santacruz) and LC3B(CST)
P62
GAPDH
Cntrl 1 6 12 24 48 (h)
LC3B
B-actin
Cntrl 10 20 µM
ATG-5
B-actin
Beclin1
GAPDH
Cntrl 10 µM
Cntrl 10 20 µM
f. Cell: HOS Drug: VP Experiment:GFP-LC3-RFP trafficking (microscopy) Software_ Zen2.3 SP1
e. Cell: HOS Drug: VP Experiment: Western Blotting Antibody:GAPDH(Santacruz) and Rab7(CST)
d. Cell: HOS Drug: VP and CQ Experiment: MDC Fluorimetry
VP
Cntrl
Rab7
GAPDH
 Cntrl 1 6 24 48 (h)

## Slide 3
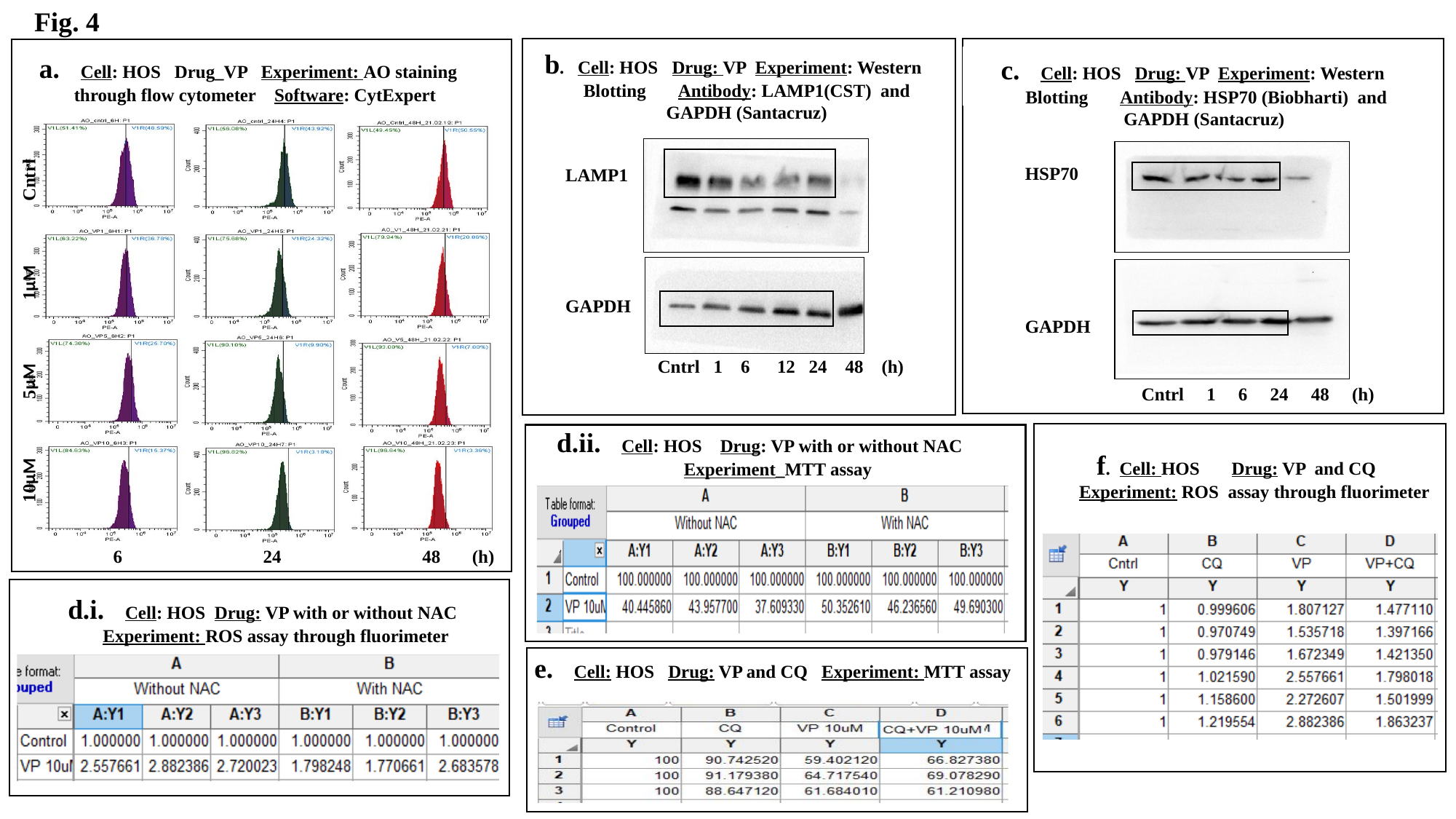

Fig. 4
b. Cell: HOS Drug: VP Experiment: Western Blotting Antibody: LAMP1(CST) and GAPDH (Santacruz)
a. Cell: HOS Drug_VP Experiment: AO staining through flow cytometer Software: CytExpert
c. Cell: HOS Drug: VP Experiment: Western Blotting Antibody: HSP70 (Biobharti) and GAPDH (Santacruz)
HSP70
GAPDH
LAMP1
GAPDH
Cntrl 1 6 12 24 48 (h)
 Cntrl 1 6 24 48 (h)
10µM 5µM 1µM Cntrl
d.ii. Cell: HOS Drug: VP with or without NAC Experiment_MTT assay
f. Cell: HOS Drug: VP and CQ Experiment: ROS assay through fluorimeter
6 24 48 (h)
d.i. Cell: HOS Drug: VP with or without NAC Experiment: ROS assay through fluorimeter
e. Cell: HOS Drug: VP and CQ Experiment: MTT assay

## Slide 4
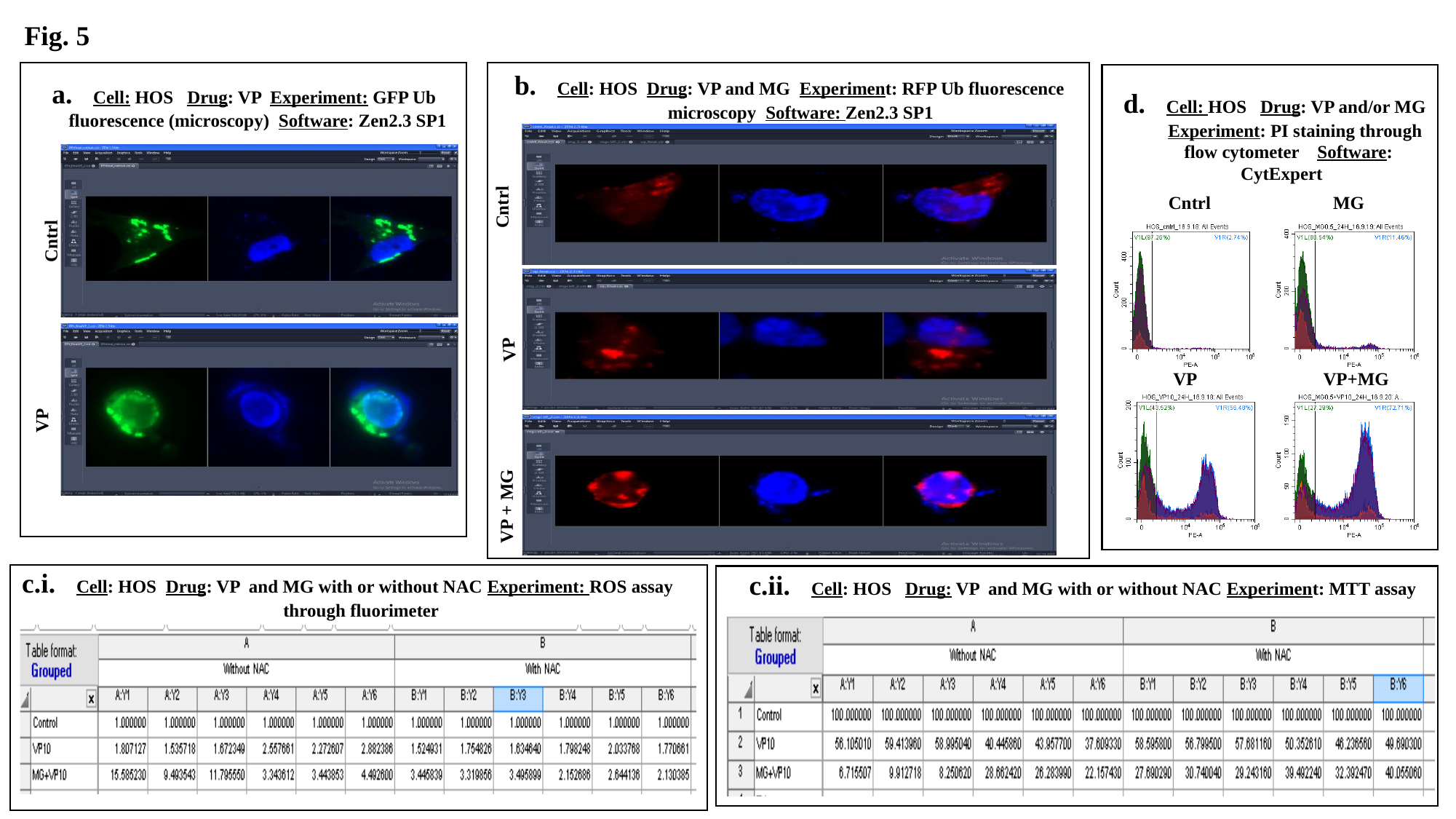

Fig. 5
b. Cell: HOS Drug: VP and MG Experiment: RFP Ub fluorescence microscopy Software: Zen2.3 SP1
a. Cell: HOS Drug: VP Experiment: GFP Ub fluorescence (microscopy) Software: Zen2.3 SP1
d. Cell: HOS Drug: VP and/or MG Experiment: PI staining through flow cytometer Software: CytExpert
MG
Cntrl
Cntrl
Cntrl
VP
VP
VP+MG
VP
VP + MG
c.i. Cell: HOS Drug: VP and MG with or without NAC Experiment: ROS assay through fluorimeter
c.ii. Cell: HOS Drug: VP and MG with or without NAC Experiment: MTT assay

## Slide 5
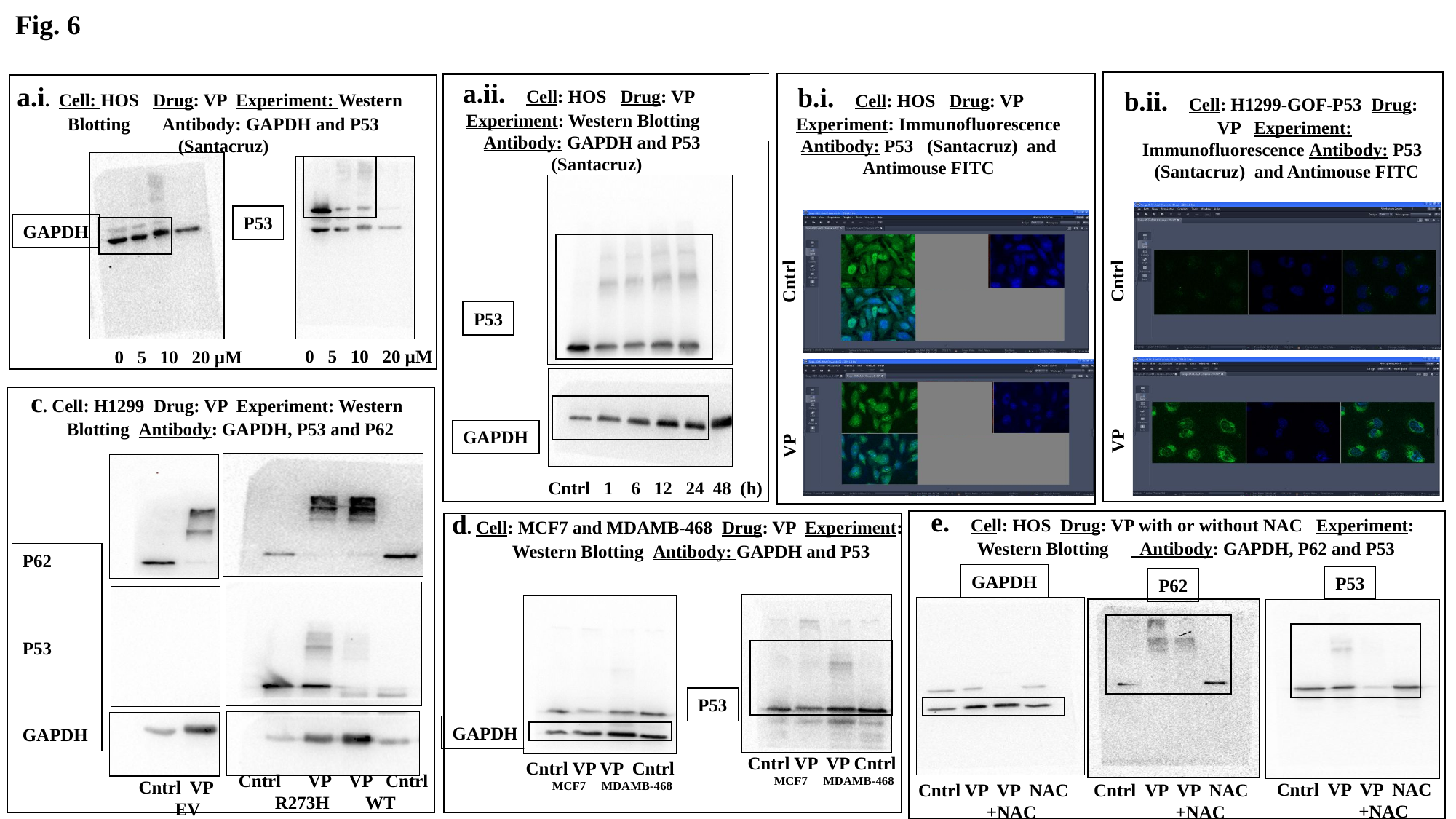

Fig. 6
a.ii. Cell: HOS Drug: VP Experiment: Western Blotting Antibody: GAPDH and P53 (Santacruz)
a.i. Cell: HOS Drug: VP Experiment: Western Blotting Antibody: GAPDH and P53 (Santacruz)
b.i. Cell: HOS Drug: VP Experiment: Immunofluorescence Antibody: P53 (Santacruz) and Antimouse FITC
b.ii. Cell: H1299-GOF-P53 Drug: VP Experiment: Immunofluorescence Antibody: P53 (Santacruz) and Antimouse FITC
P53
GAPDH
Cntrl
Cntrl
P53
0 5 10 20 µM
0 5 10 20 µM
c. Cell: H1299 Drug: VP Experiment: Western Blotting Antibody: GAPDH, P53 and P62
GAPDH
VP
VP
Cntrl VP VP Cntrl
 R273H WT
Cntrl VP
 EV
Cntrl 1 6 12 24 48 (h)
e. Cell: HOS Drug: VP with or without NAC Experiment: Western Blotting Antibody: GAPDH, P62 and P53
d. Cell: MCF7 and MDAMB-468 Drug: VP Experiment: Western Blotting Antibody: GAPDH and P53
P53
GAPDH
P62
P53
GAPDH
GAPDH
P53
P62
 Cntrl VP VP Cntrl
 MCF7 MDAMB-468
 Cntrl VP VP Cntrl
 MCF7 MDAMB-468
 Cntrl VP VP NAC
 +NAC
 Cntrl VP VP NAC
 +NAC
 Cntrl VP VP NAC
 +NAC

## Slide 6
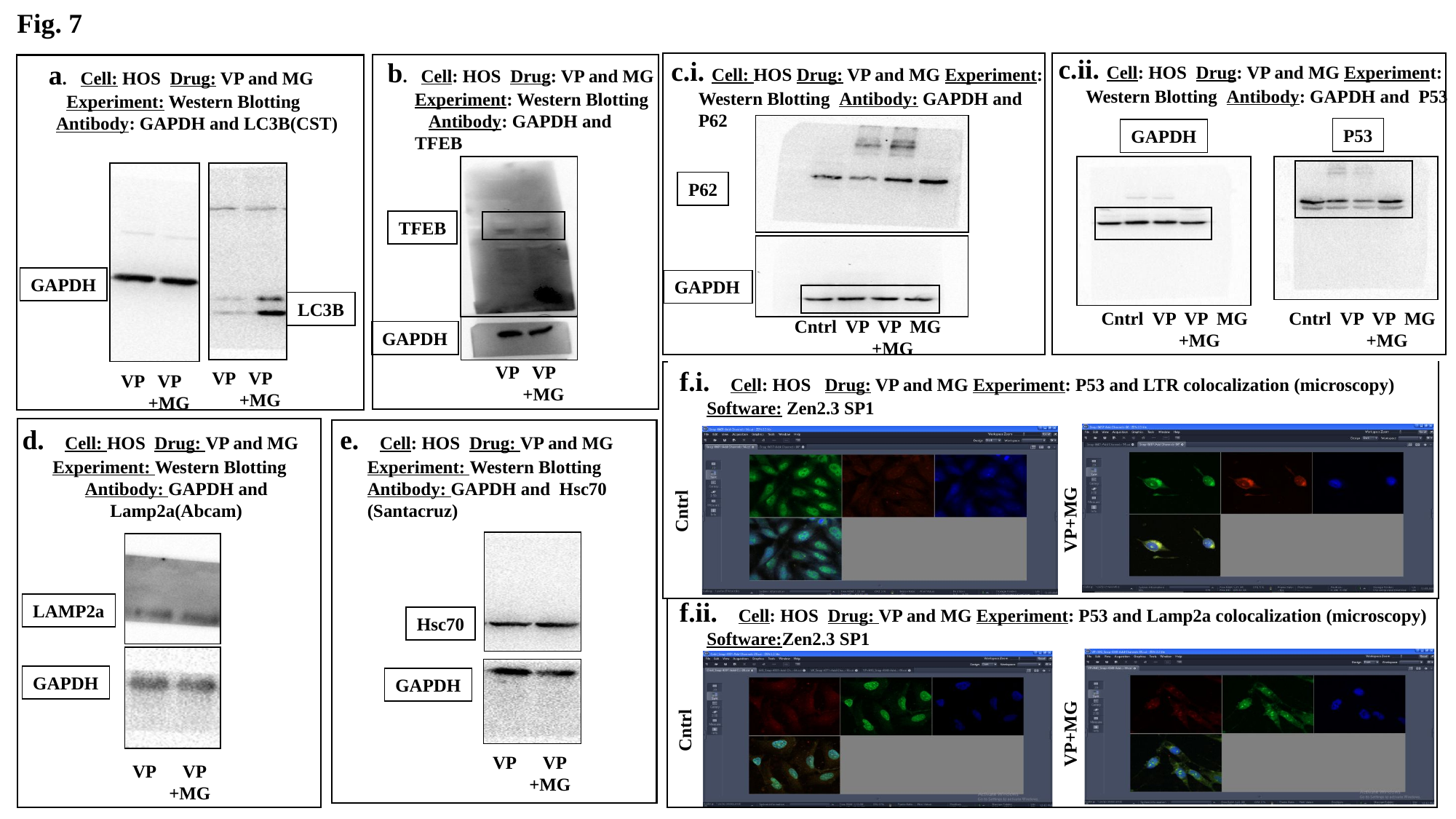

Fig. 7
c.ii. Cell: HOS Drug: VP and MG Experiment: Western Blotting Antibody: GAPDH and P53
c.i. Cell: HOS Drug: VP and MG Experiment: Western Blotting Antibody: GAPDH and P62
b. Cell: HOS Drug: VP and MG Experiment: Western Blotting Antibody: GAPDH and TFEB
a. Cell: HOS Drug: VP and MG Experiment: Western Blotting Antibody: GAPDH and LC3B(CST)
P53
GAPDH
TFEB
GAPDH
LC3B
P62
GAPDH
Cntrl VP VP MG
 +MG
Cntrl VP VP MG
 +MG
Cntrl VP VP MG
 +MG
GAPDH
VP VP
 +MG
f.i. Cell: HOS Drug: VP and MG Experiment: P53 and LTR colocalization (microscopy) Software: Zen2.3 SP1
VP VP
 +MG
VP VP
 +MG
d. Cell: HOS Drug: VP and MG Experiment: Western Blotting Antibody: GAPDH and Lamp2a(Abcam)
e. Cell: HOS Drug: VP and MG Experiment: Western Blotting Antibody: GAPDH and Hsc70 (Santacruz)
Cntrl
VP+MG
f.ii. Cell: HOS Drug: VP and MG Experiment: P53 and Lamp2a colocalization (microscopy) Software:Zen2.3 SP1
LAMP2a
Hsc70
GAPDH
GAPDH
Cntrl
VP+MG
VP VP
 +MG
VP VP
 +MG

## Slide 7
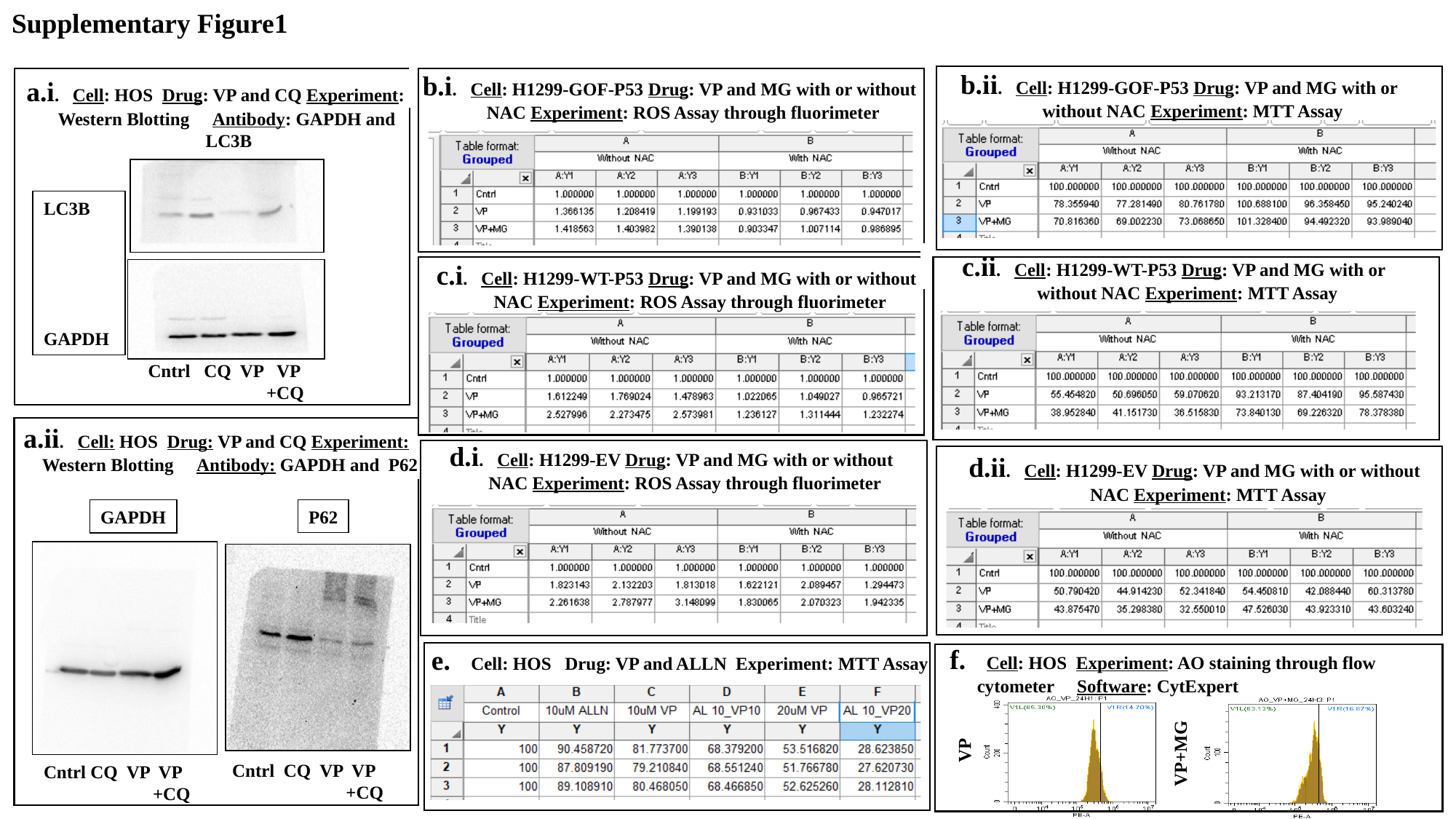

Supplementary Figure1
b.ii. Cell: H1299-GOF-P53 Drug: VP and MG with or without NAC Experiment: MTT Assay
b.i. Cell: H1299-GOF-P53 Drug: VP and MG with or without NAC Experiment: ROS Assay through fluorimeter
a.i. Cell: HOS Drug: VP and CQ Experiment: Western Blotting Antibody: GAPDH and LC3B
LC3B
GAPDH
c.ii. Cell: H1299-WT-P53 Drug: VP and MG with or without NAC Experiment: MTT Assay
c.i. Cell: H1299-WT-P53 Drug: VP and MG with or without NAC Experiment: ROS Assay through fluorimeter
Cntrl CQ VP VP
 +CQ
a.ii. Cell: HOS Drug: VP and CQ Experiment: Western Blotting Antibody: GAPDH and P62
d.i. Cell: H1299-EV Drug: VP and MG with or without NAC Experiment: ROS Assay through fluorimeter
d.ii. Cell: H1299-EV Drug: VP and MG with or without NAC Experiment: MTT Assay
P62
GAPDH
f. Cell: HOS Experiment: AO staining through flow cytometer Software: CytExpert
e. Cell: HOS Drug: VP and ALLN Experiment: MTT Assay
VP
VP+MG
Cntrl CQ VP VP
 +CQ
Cntrl CQ VP VP
 +CQ
